# Mining biological information from 3D short time-series gene expression data: the OPTricluster algorithm

**DOI:** 10.1186/1471-2105-13-54

**Published:** 2012-04-04

**Authors:** Alain B Tchagang, Sieu Phan, Fazel Famili, Heather Shearer, Pierre Fobert, Yi Huang, Jitao Zou, Daiqing Huang, Adrian Cutler, Ziying Liu, Youlian Pan

**Affiliations:** 1 Knowledge Discovery Group, Institute for Information Technology, National Research Council Canada, 1200 Montréal Road, Ottawa, ON K1A 0R6, Canada; 2 Seed Systems Group, Plant Biotechnology Institute, 110 Gymnasium Place, Saskatoon, SK S7N 0W9, Canada; 3 Oil Crops Research, Institute, Chinese Academy of Agricultural Sciences, No. 2 Xudong 2nd Road, Wuhan, Hubei 430062, China

## Abstract

**Background:**

Nowadays, it is possible to collect expression levels of a set of genes from a set of biological samples during a series of time points. Such data have three dimensions: gene-sample-time (GST). Thus they are called 3D microarray gene expression data. To take advantage of the 3D data collected, and to fully understand the biological knowledge hidden in the GST data, novel subspace clustering algorithms have to be developed to effectively address the biological problem in the corresponding space.

**Results:**

We developed a subspace clustering algorithm called Order Preserving Triclustering (OPTricluster), for 3D short time-series data mining. OPTricluster is able to identify 3D clusters with coherent evolution from a given 3D dataset using a combinatorial approach on the sample dimension, and the order preserving (OP) concept on the time dimension. The fusion of the two methodologies allows one to study similarities and differences between samples in terms of their temporal expression profile. OPTricluster has been successfully applied to four case studies: immune response in mice infected by malaria (*Plasmodium chabaudi*), systemic acquired resistance in *Arabidopsis thaliana*, similarities and differences between inner and outer cotyledon in *Brassica napus *during seed development, and to *Brassica napus *whole seed development. These studies showed that OPTricluster is robust to noise and is able to detect the similarities and differences between biological samples.

**Conclusions:**

Our analysis showed that OPTricluster generally outperforms other well known clustering algorithms such as the TRICLUSTER, gTRICLUSTER and K-means; it is robust to noise and can effectively mine the biological knowledge hidden in the 3D short time-series gene expression data.

## Background

Clustering of co-expressed genes has been an active data mining topic and advanced in parallel with the development of microarray technology [1]. There is a vast amount of literature on clustering algorithms developed for microarray data analysis [1]. Microarray gene expression data can be classified into two categories: steady state and time-series gene expression data [2]. Time-series gene expression data are widely used to study the dynamic behaviour of various biological processes in the cell [3-5]. They can be classified into two categories (relative to the clustering algorithms design for their analysis): short time-series corresponding to 3-8 time points [6], and long time-series corresponding to more than 8 time points. Short time-series are the most abundant type of time-series data in the literature [6]. Short-time series data are usually very noisy. Algorithms that are designed to analyze either steady state data or long time-series data do not perform well on short time-series data due to their relatively small number of time points [5-7]. Hence, it is necessary to develop algorithms that can be used specifically for their analysis.

Pioneering clustering algorithms such as K-means [8], Hierarchical clustering [9], and Self Organizing Map [10] identify full space clusters. Unfortunately, in many applications, subspace clusters are more meaningful than full space clusters [11]. Biclustering algorithms were recently proposed to find subgroups of genes that exhibit similar behaviour across subsets of samples, experimental conditions, or time points [11-15]. Nowadays, it is possible to collect expression levels of a set of genes for a given set of biological samples during a series of time points. Such data have three dimensions, gene-sample-time (GST), and thus are called 3D gene expression data. To take advantage of the 3D data collected, and to fully understand the biological knowledge hidden in the GST data, we have to move beyond the full space clustering concepts and develop algorithms that can effectively address the problem in the corresponding 3 dimensions [16-18]. A 3D cluster consists of a subset of genes of similar expression profiles along a segment of time-series, in a subset of samples. This kind of coherent clusters may contain information that could be used to identify useful phenotypes, potential genes related to these phenotypes and their interaction/regulation.

Although subspace clustering algorithms are biologically more meaningful than full space clustering algorithms, the identification of full space clusters are less costly compared to subspace clusters. In fact, most subspace clustering algorithms have been shown to be NP-complete [11], thus making their identification computationally expensive. This is due to the fact that in full space clustering, one usually looks for clusters across the entire dataset at once, whereas in subspace clustering one looks for clusters across all possible subsets of the data space.

Most clustering models, including those used in subspace clustering described above, define similarity among different objects by *distances *over either all or only a subset of the dimensions [1]. However, distance functions, such as Euclidean distance, Manhattan distance, and cosine distance are not always adequate in capturing correlations among the objects. In fact, strong correlations may still exist among a set of objects even if they are far apart from each other as measured by the distance functions [1]. Pioneering works on triclustering algorithm relied on graph-based approaches and similarity measures to mine triclusters [16-18]. For example, the triclustering algorithm of [16] mines the largest triclusters satisfying a constant multiplicative or additive relationship between the expression levels in a cluster. Such a strict constraint considerably limits the capability of an algorithm to identify useful patterns and may not be able to fully cope with noise when dealing with time-series gene expression data in general. Several of the algorithms designed for the analysis of long time-series - do not work well on short time-series due to over fitting [6]. Also, most pioneering triclustering algorithms described in the literature only focus on the similarities between the biological samples and do not consider differences between them.

In this paper, we developed an order preserving 3D clustering algorithm named OPTricluster, for 3D short time-series data mining. A 3D short time series corresponds to GST with 2-5 samples and 3-8 time points. In fact most of the 3D time series gene expression data in the GEO database [19] have less than 5 samples and less than 9 time points. OPTricluster is able to identify triclusters (3D clusters) with coherent evolution from a given 3D dataset using a combinatorial approach on the sample dimension, and the order preserving (OP) concept on the time dimension. We say that a matrix is order preserving if there exists a permutation of its columns such that its rows are monotonic functions [13]. The OP concept is a well established mathematical theory, and has been used by several other authors in the past to tackle the problem of gene expression data analysis [13,14,20,21].

The integration of the two methodologies (combinatorial and OP) allows one to study similarities and differences between samples in terms of temporal expression profile and/or differential expression. Basically, the novel triclustering algorithm is able to mine triclusters of genes in a subset of samples, with expression level having same directions (i.e. increase, decrease and/or stay constant similarly) across the time-series experiments. OPTricluster takes into account the sequential nature of the time-series and the noisy nature of the data through the OP concept. The OP model focuses on the similarity in the relative order of the time points, rather than on the distance between the actual expression levels as in several other clustering models [7-10]. This approach is potentially more robust to the stochastic nature of the gene expression levels, and to the variation caused by the measurements procedures.

## Results

OPTricluster is applied to analyze four different 3D gene expression datasets: immune response in mice infected by malaria (*Plasmodium chabaudi*), systemic acquired resistance (SAR) in *Arabidopsis thaliana*, similarities and differences between inner and outer cotyledon in *Brassica napus *during seed development, and *Brassica napus *whole seed development. We used these four datasets to show how OPTricluster can be used to tackle a variety of problems in Computational Biology. For example, application of OPTricluster to the mouse dataset shows how it can be used to study similarities and differences between biological samples in terms of temporal expression profiles. Application to the *Arabidopsis thaliana *SAR dataset shows how it can be used not only to study similarities and differences between biological samples, but also to infer the transcription network, that is, the relationship between the transcription factors (TFs) and their target genes. Application to the *Brassica napus *cotyledon dataset shows how OPTricluster can be used to study the spatial similarities and differences between biological samples in term of temporal expression profile, whereas its application to the *Brassica napus *whole seed dataset shows how OPTricluster can be used to handle classical short time-series dataset (2 dimensional dataset).

### Implementation

OPTricluster is implemented entirely in Java and will work with any operating system supporting Java 1.6 or later. The executable OPTricluster Java package is available as Additional File 1, and its user manual as Additional File 2. Portions of the interface of OPTricluster are implemented using a third party library, the JFreeChart [22]. In a post processing step, OPTricluster also makes use of external Gene Ontology files. OPTricluster can download the Gene Ontology and gene annotation files directly from the Gene Ontology websites. In fact the GO analysis plug-in of the Gene Ontology Analysis (GOAL) [23] package that we recently developed is integrated into OPTricluster for biological evaluation of the clusters. A user of OPTricluster first specifies a tab delimited gene expression data file as input to OPTricluster. The file is uploaded and displayed as a table on the screen. The user can check their data and do other manipulations on the data once it is displayed. This includes visual check, plot and view the distribution of the input gene expression data, which may help in deciding the best ranking threshold input parameter (see Methodology Section). Next, the user selects the analysis parameters through the OPTricluster parameter input interface. Following this input phase, the OPTricluster executes and a new table will appear displaying the clustering results, where new columns are added to the initial table and they correspond to the ranking profile of each gene across experimental time points in each sample respectively. From this step, the user will either select between constant, conserved, and divergent patterns for further analysis as explained below.

**Figure 1:**
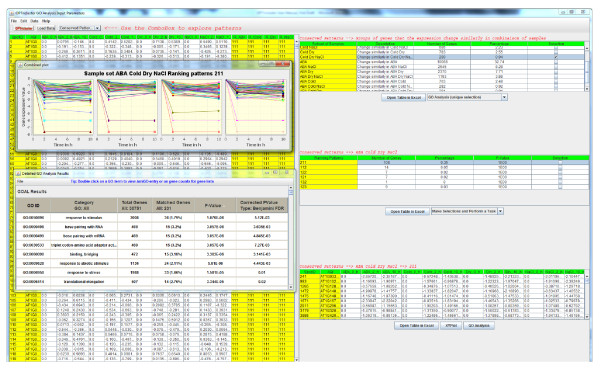
**OPTricluster software**. Example of OPTricluster interface showing a conserved cluster and its Gene Ontology analysis.

There are three types of patterns that the user can choose from: conserved patterns, divergent patterns, and constant patterns. Conserved patterns correspond to group of genes having same behaviour across experimental time points in subsets of samples. Divergent patterns correspond to group of genes that behave differently in at least one sample along the time point experiments. Constant patterns correspond to groups of genes that the expression levels do not change across experimental time points. Once the type of patterns is selected, a new table appears on the screen, describing the subset of samples the number of genes with the selected patterns in each subset of samples. By clicking on a subset of sample, another table appears showing how the genes are grouped in the selected patterns and in the corresponding subset of samples. Finally the user can view the genes in each group as a table by clicking on the corresponding ranking profile. Figure 1 shows an example of the analysis of a conserved pattern. We refer the reader to the OPTricluster user manual (Additional File 2) for more details relative to OPTricluster Software.

Note that, at each step of the analysis described above, OPTricluster offers the possibility to the user to perform several other tasks and gain more knowledge on the results obtained at the corresponding step. These manipulations include: open the table in Excel and use the Excel capabilities for other manipulations. Plot and view the pie chart or the bar chart of the results. Merge cluster with similar profile for further analysis. Obtain the difference between patterns in different subsets of samples. Perform and visualize the gene ontology enrichment analysis of the group of genes obtained at that particular step.

### Simulation and robustness to noise

To test the robustness of OPTricluster to noise, we used the adjusted rand index (ARI). ARI has been used previously for clustering techniques comparison and robustness to noise [18]. ARI's values lie between 0 and 1, where larger value means higher similarity between the clustering results. If the experimental result is perfectly consistent with the domain knowledge (known triclusters embedded in the simulated dataset), the index value will be 1. If a clustering is no more than a random choice, the index will be 0.

**Figure 2:**
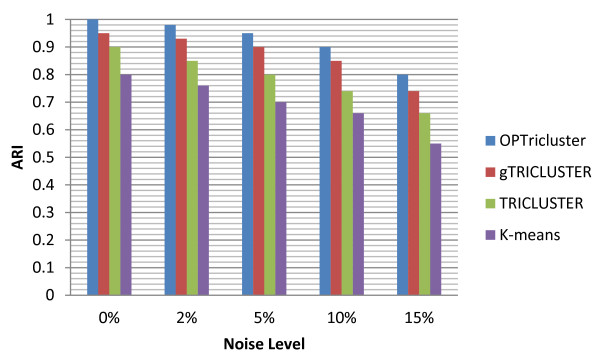
**Comparative analysis of clustering algorithms**. Simulation results showing the comparative evaluation of OPTricluster, the modified gTRICLUSTER, modified TRICLUSTER, and the K-means algorithm.

We generated a synthetic 3D microarray dataset, consisting of N = 1000 genes, M = 4 samples, and L = 3 time points, with four order preserving triclusters across the entire four samples imbedded in it. Each tricluster had 20, 25, 30, and 100 genes respectively and there was no overlap between the four triclusters. Thus the domain knowledge corresponds to the four embedded triclusters. We added 0%, 2%, 5%, 10%, and 15% noise to the original dataset and computed the ARI values between the triclustering results on noisy datasets and the domain knowledge. Such a process was performed using OPTricluster a modified version of TRICLUSTER [16] gTRISCLUSTER [18], and the K-means algorithm. For the K-means algorithm the 3D dataset is converted into a 2D dataset as follows. The N × M × L dataset becomes N × ML dataset. For each level of noise, each algorithm was run five times and the mean of the ARI obtained were represented in Figure 2. It can be seen that the ARI values of OPTricluster are larger than those of modified gTRICLUSTER, modified TRICLUSTER, and the K-means algorithm. This means that the OPTricluster algorithm developed in this study performs better on order preserving patterns, and it is more robust to noise than modified TRICLUSTER, modified gTRICLUSTER, and the K-means algorithm. Comparative analyses were only performed by studying similarity (same OP patterns) between samples because TRICLUSTER, gTRICLUSTER, and the K-means algorithm cannot investigate differences between samples, for which, only the proposed OPTricluster does.

### Immune responses in mice infected by malaria

The goal of this study is to examine whether immune responses to malaria (*Plasmodium chabaudi*) infection differ between the sexes and are altered by the presence of gonadal steroids. To tackle this problem, we used a 3D short time-series gene expression dataset downloaded from the Gene Expression Omnibus website [19], (accession number: GSE4324). This dataset has N = 33935 probes and it corresponds to the gene expression profiles of mice response to *Plasmodium chabaudi *[24], representing two disease states (*P. chabaudi *infected and non-infected), two genders (male and female), two protocols (intact and gonadectomized), and four time point experiments: 0, 3, 7, and 14 days after inoculation (DAI). We refer to the four biological samples used in this study as follows: intact male (*I_M_*), intact female (*I_F_*), gonadectomized male (*G_M_*), and gonadectomized female (*G_F_*). For ethical issue pertaining to this dataset, see reference [24].

After data pre-processing and normalization, we ended up with 5783 significant probes, corresponding to 5063 unique genes. The three dimensions of the data are: G (N = 5783 probes), S (M = 4 samples: *I_M_*, *I_F_*, *G_M_*, and *G_F_*), and T (L = 4 time points: 0, 3, 7, and 14 DAI). We set the input parameters to: minimum number of genes in a cluster *I_m _= *1, minimum number of samples in a cluster *J_m _= *1, and differential expression threshold (or ranking threshold) *δ = *0.31. With the minimum number of samples in a tricluster set to 1, the algorithm generated 2^4^-1 = 15 combinations of samples ({*I_M_, I_F_, G_M_, G_F_*}, {*I_M_, I_F_, G_M_*}, {*I_M_, I_F_, G_F_*}, {*I_M_, G_M_, G_F_*}, {*I_F_, G_M_, G_F_*}, {*I_M_, I_F_*}, {*I_M_, G_M_*}, {*I_M_, G_F_*}, {*I_F_, G_M_*}, {*I_F_, G_F_*}, {*G_M_, G_F_*}, {*I_M_*}, {*I_F_*}, {*G_M_*}, {*G_F_*}).

**Figure 3:**
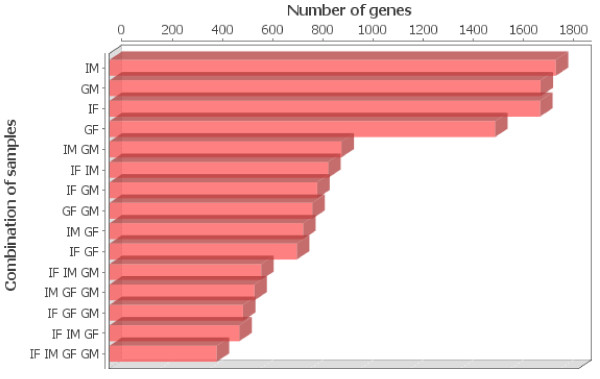
**Similarities among samples in mouse data**. Similarities in the gene expression data of mice response to *Plasmodium chabaudi *infection. The x-axis corresponds to the subset of samples and the y-axis the number of genes that behave similarly in this subset of samples.

**Figure 4:**
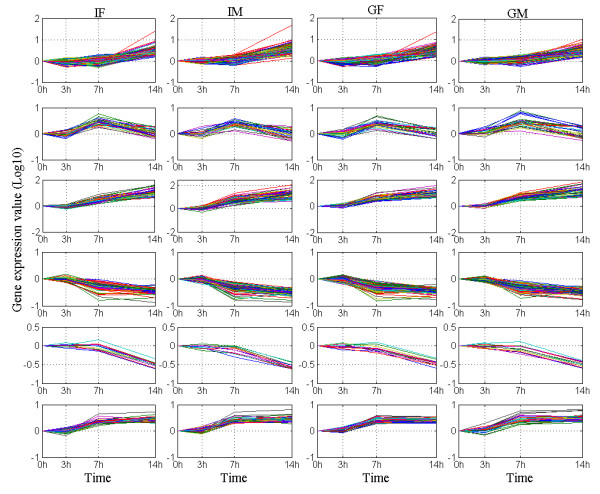
**Example of similar patterns in mouse data**. Set of genes with conserved expression profiles across the four biological samples: {*I_F_, I_M_, G_F_, G_M_*}. The x-axis corresponds to the experimental time points and the y-axis to the expression level of the genes across the time series and in the four samples. Note that each sample corresponds to the column chart.

**Figure 5:**
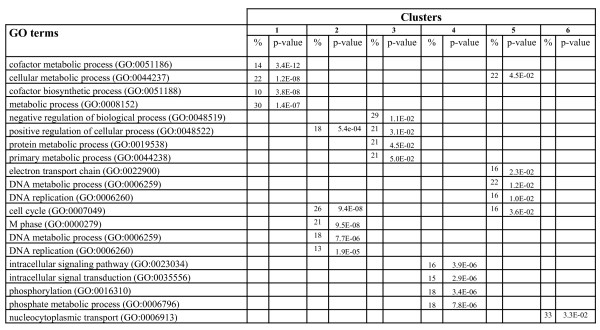
**Gene Ontology analysis of mouse clusters**. GO analysis of the 6 clusters that are affected similarly in the four biological samples {*I_M_, I_F_, G_M_, G_F_*}.

Figure 3 shows the set of genes in which the expression level changed similarly by the infection across the time series, in one and a combination of two or more samples. Among the 3943 probes conserved in the four biological samples {*I_M_, I_F_, G_M_, G_F_*}, 3516 genes are unchanged, whereas 427 (Figure 3) changed similarly in all four samples. These 427 genes are further clustered into six groups (Figure 4) with 12 or more genes. Clearly, the genes in Figure 4 have similar behaviour in the four samples and across the entire time series. Most of these 427 genes may play the role of housekeeping. In other words, they represent the set of genes that are co-expressed regardless of the experimental condition to maintain basic cellular function. Indeed, Gene Ontology (GO) analysis of these six clusters (Figure 5), showed that they are involved in similar molecular function and biological processes, such as protein and DNA binding, transcription regulation, cell cycle and basic metabolism.

**Figure 6:**
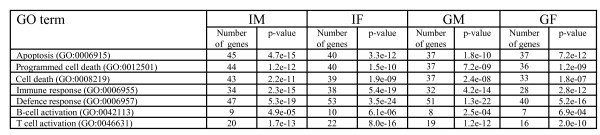
**Gene Ontology analysis of genes affected in Mouse**. GO analysis of the genes affected in each sample after *Plasmodium *infection.

The results shown in Figure 3 suggest that intact males (*I_M_*) have the highest number of genes (1778) that the expression level changed following pathogen attack. This indicates that *I_M _*is probably more vulnerable to *Plasmodium *infection compared to the other three phenotypes. This is consistent with the phenotypical observation made in [24]. Indeed the Gene Ontology analysis shows that *I_M _*have more genes involved in the GO biological processes, such as, cell death (GO:0008219), programmed cell death (GO:0012501), apoptosis (GO:0006915), than *I_F _*(Figure 6). On the other hand, these same results showed that gonadectomy of males altered the sex-associated differences, suggesting that sex steroid hormones may modulate immune responses to infection [24].

In terms of differences between the four samples tested, our analysis identified genes that were unique to only one and a combination of two or more samples. For example we identified 251, 266, 216, and 249 genes unique to *I_M_*, *I_F_*, *G_M_*, and *G_F _*respectively. These genes may be the origin of the differences between the four samples after *Plasmodium *infection. Thus, they may represent potential targets or biomarkers to be used not only to understand the differences between the samples, but also to develop novel therapeutic means.

### Systemic acquired resistance in *Arabidopsis thaliana*

We applied OPTricluster to study similarities and differences in defence mechanism of *Arabidopsis thaliana *against pathogenesis. The goal of the study is to understand the roles of NPR1 and some of TGA family TFs during systemic acquired resistance in *Arabidopsis*. The 3D microarray data used here was obtained using Affymetrix *Arabidopsis *Genechip consisting of 22810 probes. The Columbia wild-type (W), mutant *npr1 *(P), double mutant *tga1 tga4 *(Z_1_), and triple mutant *tga2 tga5 tga6 *(Z_2_) were treated with salicylic acid (SA) for 0, 1, and 8 hours. After data pre-processing and normalization, we ended up with 3945 significant genes. We set the Columbia wild-type as our baseline and took the log_2 _ratio of the mutant gene expression levels over the wild-type at respective time points.

**Figure 7:**
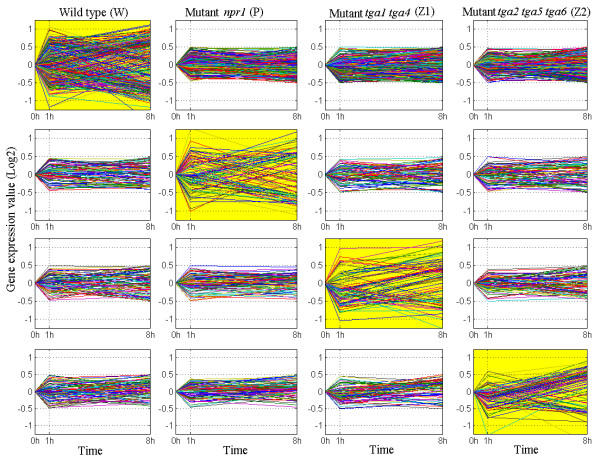
**Example of Divergent Patterns**. Example of genes that may be specific to the W, P, Z_1_, and Z_2 _respectively. The x-axis corresponds to the experimental time points and the y-axis to the expression level of the genes across the time series and in the four samples. Note that each sample corresponds to the column chart: W, P, Z_1_, and Z_2 _respectively.

Given the *N × M × L *gene expression matrix, our goal is to identify the set of genes that are controlled by the TFs at a given time point, to study similarities and differences between them, and to infer a temporal transcriptional regulatory network controlling SAR in *A. thaliana*. OPTricluster generated 2^4^-1 = 15 combinations of samples. Below we present some of the results obtained by OPTricluster. Figure 7 for example shows an example of divergent patterns. The expression levels of these genes are relatively unchanged (constant) in three samples and behave differently in one. For example, in the first row, significant changes are visible within WT, whereas the other three genotypes stay relatively constant (within threshold of ± 0.5) across the three time point. Clearly, since the expression level of these genes stay constant in three of the four experimental conditions and change considerably in only one of them, these genes may represent potential targets of the TFs tested in this study.

**Figure 8:**
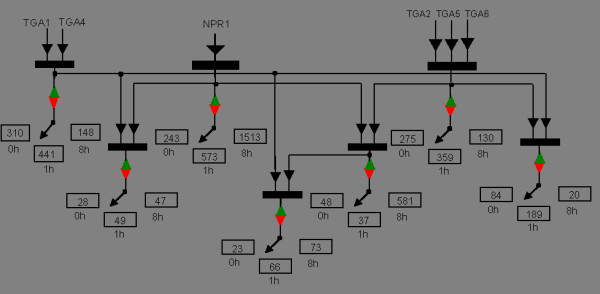
**SAR transcription networks**. SAR transcriptional network at 0, 1, and 8 h. In the diagrams, ▬ = action could be inclusive or, **▼ **= information flow, red inverted triangle symbol = up regulation, and green triangle symbol = down regulation. Numbers in boxes represent the number of genes regulated by the corresponding groups of TFs at respective time points.

Figure 8 shows a wiring diagram of the genetic network of SAR in *Arabidopsis thaliana *at 0 h, 1 h, and 8 h inferred using the OPTricluster algorithm (Equation 4 in the Method Section). For example, our analysis showed that only 23, 66, and 73 genes are either down- or up-regulated by the combined action of the three sets of TFs at 0, 1, and 8 h respectively. The number of NPR1 targeted genes is less than that of TGA1 TGA4 and TGA2 TGA5 TGA6 at 0 h. But at 8 h, it is the reverse situation where the number of NPR1 targeted genes is higher than those regulated by TGA1 TGA4 and TGA2 TGA5 TGA6, respectively. This is consistent with the fact that NPR1 gene expression in the Columbia wild type was initially moderate but drastically increased at 1 hour and increased further until 8 hours after SA treatment (Additional file 3).

Gene Ontology analysis reveals that several of the genes that are regulated by the three sets of TFs (NPR1, TGA1 TGA4, and TGA2 TGA5 TGA6) at 8 h (Figure 8) are associated with response to stimulus (GO:0050896; p-value = 1.1e-08), stress (GO:0006950; p-value = 1.7e-05), abiotic stimulus (GO:0009628; p-value = 3.1e-05), biotic stimulus (GO:0009607; p-value = 1.3e-03), and defence mechanism (GO:0006952; p-value = 5.0e-02). These correspond to the fact that the plants were treated by SA, which mimic pathogen infection. They also confirm the fact that the TFs tested in this study are known to play major roles in plant defence mechanism [25]. In this application of OPTricluster, we used similarities in gene expression profiles of *Arabidopsis thaliana *with single, double or triple mutations of key transcription factors in the defence signalling network. We studied the network dynamics over a time series after treatment with salicylic acid (SA), which mimics a pathogen infection. We found that most SA-responsive genes were affected by at least one mutation and that most affected genes fit one of a few patterns of regulation. We then provided a first glimpse into the temporal pattern of the gene regulatory network during systemic acquired resistance in *Arabidopsis*.

### Similarities and differences between inner and outer cotyledon in *brassica napus *during seed development

Canola (*Brassica napus*) has two cotyledons; one embraces the other. The outer cotyledon is bigger and has higher oil content than the inner one. There exists similarity and differences in the development of and the metabolic processes between the two cotyledons. The objective of this analysis is to unravel the spatial similarity and differences in gene expression profiles between the two cotyledons in order to identify genes that play important roles in seed development and oil production. After obtaining the respective cotyledons, DNA microarray experiments were performed on each cotyledon using the DNA Combimatrix 90 K chip, developed by Plant Biotechnology Institute, NRC [26]. The output yielded two time-series data matrices *X_I _*and *X_O _*representing the gene expression level in the inner and outer cotyledons, respectively. The time-series has six time points, 20, 22, 24, 26, 28, and 30 days after pollination (DAP). After data pre-processing and normalization, significantly expressed genes were identified for this study. The three dimensions considered in this analysis were G (N = 3945 genes), S (M = 2 biological samples: *I *and *O*), and T (L = 6 time points: 20, 22, 24, 26, 28, and 30 DAP).

**Figure 9:**
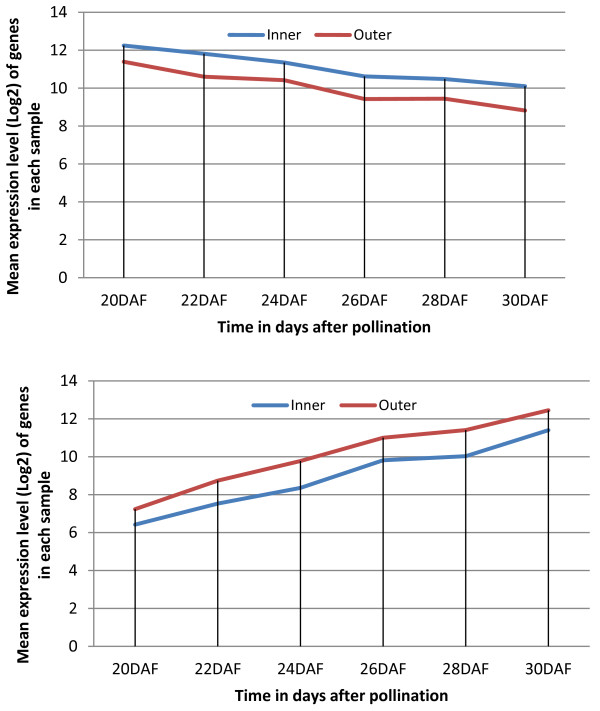
**Differences between inner and outer cotyledons**. Gene expression differences between inner and outer cotyledons. The x-axis corresponds to the experimental time points and the y-axis to the mean of the expression level of genes in each sample. Each line represents a sample.

With the minimum number of samples in a tricluster set to 1 and a threshold of ~ 1.5 fold change, the algorithm generated 2^2^-1 = 3 combinations of samples ({*I, O*}, {*I*}, {*O*}*)*. The subset of sample {*I, O*} yielded similar patterns between the inner and outer cotyledons, whereas the equations {*O*} *- *{*I, O*}, or {*I*} *- *{*I, O*} yielded patterns specific to outer or inner cotyledon, respectively. Analysis reveals 33 genes depicting the main difference between the two samples across the six time point experiments (Figure 9) and several others across subsets of the six time points (Additional file 4). Among the 33 genes, 17 and 16 were highly expressed in inner compared to outer, and outer compared to inner, respectively.

**Figure 10:**
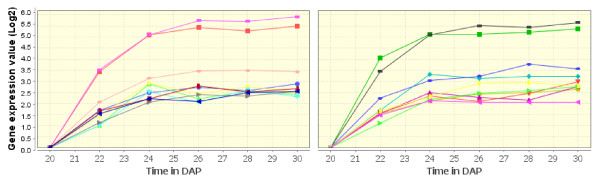
**Example of conserved patterns in the cotyledon datasets**. Example of group of genes with similar expression profile in the inner and the outer cotyledons dataset.

Figure 9 shows that the expression level of the genes that are highly expressed in the inner compared to the outer cotyledon decreases with time whereas the ones that are highly expressed in the outer compare to the inner cotyledon increase with time. In general, in terms of the direction of the gene expression profile (Figure 10 for example), OPTricluster did not depict significant differences between the inner and the outer cotyledons. The main difference between the inner and the outer cotyledons resides in the amplitude of the gene expression level.

### *Brassica napus *whole seed development

**Figure 11:**
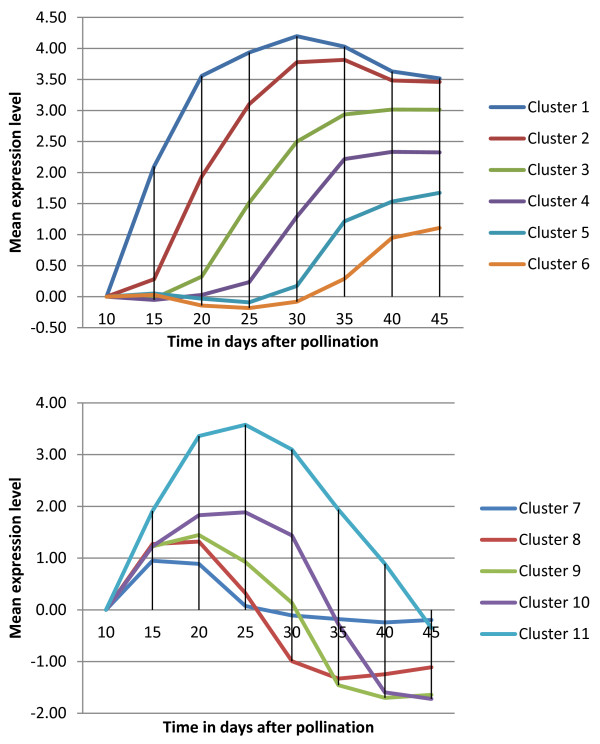
**Whole seed Brassica napus clusters**. Example of patterns identified in *Brassica napus *whole seed dataset using OPTricluster. The x-axis corresponds to the experimental time points whereas the y-axis to the mean of the expression level of the genes in each cluster. Each curve corresponds to a cluster.

We also analyzed another in house Combimatrix 94 K *Brassica napus *microarray dataset, which corresponds to the gene expression profiles during seed development. It has eight times points: 10, 15, 20, 25, 30, 35, 40, and 45 DAP. Each time point has six biological replicates. After data pre-processing and normalization, we ended up with 10865 probes, from which we took the mean of the replicates. Figure 11 show examples of patterns identified using OPTricluster.

Biological evaluation of these clusters showed that they are highly enriched under seed development; fatty acid and lipid metabolism; fatty acid and lipid biosynthesis; lipid storage, transport, and localization; oil content and biosynthesis, with *p*-values < 1.0e-03 (Additional file 5). These results demonstrate that seed development and fatty acid synthesis are highly correlated. Indeed, this is consistent with the fact that in higher plants, the biosynthesis of most fatty acids and lipids is physiologically coupled with seed development [27]. Further analyses showed that most of these patterns are highly positively or negatively correlated with the expression profile of transcription factors such as: LEC1, WRI1, FUS3, ABI3, and ABI5. This observation is consistent with the fact that the LEC1 function is partially dependent on ABI3, FUS3, and WRI1 in the regulation of fatty acid and fatty acid derived complex lipid [27-29]. It is also consistent with the fact that WRI1 plays a significant role during oil accumulation in maturing seed, that WRI1 is a prerequisite for fatty acid synthesis, and is important for normal embryo development [30]. Most of these results suggest that, the genes involved in these patterns may represent potential targets that could be used for the genetic improvement of oil production in *B. napus*.

## Discussion

In this paper, we developed a subspace clustering algorithm OPTricluster, for 3D short time-series data mining. OPTricluster is able to identify triclusters (3D clusters) with coherent evolution from a given 3D dataset using a combinatorial approach on the sample dimension, and the OP concept on the time dimension. The amalgamation of the two methodologies, combinatorial and order preserving allows one to study similarities and differences between samples in terms of temporal expression profile and/or differential expression. The combinatorial approach on the sample dimension is necessary because the goal of the algorithm is not only to study similarities between biological samples, but also to study differences between them at the single and multiple samples level. Because of this enumerative approach, it makes OPTricluster computational heavy when one is dealing with an increasing number of samples. As a result, we have restrictions for analyzing 3D short time series genes expression data. That is 3D gene expression with ~3-8 time points and ~2-5 samples. For longer 3D time series, OPTricluster will still work, but the computational complexity of the algorithm will increase exponentially with the increase in the number of time points and/or the number of samples.

Because of this combinatorial approach in the sample dimension and the OP concept in the time dimensions, the algorithm is able to mine clusters of genes in a subset of samples, with expression level having same directions across a time-series experiment. OPTricluster takes into account the sequential nature of the time-series and the noisy nature of the data through the OP concept. The results of Gene Ontology analysis and statistical analysis show that OPTricluster is robust to noise and is able to detect the similarities and differences between samples in terms of temporal expression profile of relevant functional categories. This is due to the fact that the OP model focuses on the similarity of the relative order over the time-series, rather than on the similarity of the actual expression levels based on a distance measure as in several other clustering models. This approach is potentially more robust to the stochastic nature of the gene expression values, and to the variation caused by the measurement procedure. This is clearly demonstrated in our comparison among the four clustering methods (Figure 2). The OP model has been accepted as a biologically meaningful clustering approach, capturing the general tendency of gene expressions across a subset of conditions [5,11-14,20,21].

Furthermore, OPTricluster also offers the possibility to analyze the genes that the expression profiles change similarly across different samples for a given time point. This is easily done within the current version of the OPTricluster algorithm by simply swapping the time dimension with the sample dimension.

One relevant application of OPTricluster is the identification of genes that may play the role of housekeeping. This is revealed by the study of similarities among the samples. Indeed, several of the genes with same behaviour across all the samples and over the entire time series are usually not affected by phenotypes (Figure 4). Thus these genes may represent genes that are needed by all cells of the organism to fulfill basic cellular functions. As has been shown in the literature, housekeeping genes have many applications. They can be used as transcription and expression controls in laboratory experiments, they can be used to infer the set of basic cellular functions, and they are essential to characterizing normal and diseased states [31]. One has to keep in mind that in medicine, genetic diseases that are linked to housekeeping genes are more likely to affect multiple organs; in microbiology, housekeeping genes of pathogens play a role in enhancing virulence; and in evolutionary biology studies, housekeeping genes diverge more slowly than other genes [31]. Therefore, their detection may enhance the creation of new drug targets and can also be used for subspecies discrimination.

The study of differences among samples in terms of co-expression is among the most important applications of the OPTricluster algorithm. This feature does not exist in the other three algorithms tested in this study. In the mice response to *Plasmodium *infection for example, OPTricluster was able to identify a set of genes specific to each sample. This group of genes can be used as biomarkers for the differentiation between these samples, thus for the detection of diseases, and subsequently use as targets to design drugs that are specific to diseases at their subtype level.

In the *Arabidopsis *data, OPTricluster identified genes that may only be regulated by one TF, or a combination of two or more TFs. This kind of information can be used to identify transcription networks as we did in this analysis, and subsequently to get more insights between TFs and gene regulation. Figure 7 for example show that only a small number of genes in each or multiple mutant genotypes overlaps across different time points. This observation suggests that most of the genes that participate in SAR have impulse behaviour; different sets of genes are targeted by the corresponding transcription factors at different time points during a response to pathogen infection. On the other hand, these results show that the true behaviour of the underlying biological process is captured at different time points, with each time point containing a unique piece of information that should be integrated in order to get the whole picture underlying the signalling pathway during SAR. Therefore, studies, such as [32], that focus on a single time point to infer genetic information and generalize the results to describe the pathogen-host interaction, could miss out important information.

In the cotyledons dataset, OPTricluster depicted differences in the inner and the outer cotyledons in terms of direction and magnitude of the gene expression level, and thus yielded more biological information relative to the spatial distribution of gene expression at the molecular, cell, tissue, organ, or system level. The analysis of the *Brassica *whole seed dataset showed that OPTricluster can not only be used to study similarities and differences among samples in a 3D short time series, but also, can be used to tackle classical short time-series problems. In several of these studies OPTricluster offered more biological insights to the problem compared to prior computational approaches used to tackle similar problems.

## Conclusions

We developed a subspace clustering algorithm for 3D short time-series gene expression data analysis. The developed algorithm is used to identify statistically and biologically significant clusters from 3D gene expression data, to study similarities and differences between samples in terms of co-expression and/or differential expression. Biological and statistical results obtained showed that OPTricluster is robust to noise and is able to detect the temporal expression profile of relevant functional categories in terms of similarities and differences in samples. The OPTricluster is implemented in Java and it is available as Additional File 1 to this manuscript.

## Methodology

Our objective is to develop a subspace clustering algorithm that is able to cope with the sequential nature of a time-series, along with the noisy nature of a dataset. We also aim at developing an algorithm that can be used to identify biologically significant subspace clusters from a given 3D short time-series gene expression dataset. This would allow one to study similarities and differences between two or more biological samples in terms of temporal expression profile.

### Definitions

Given a set of *N *genes *G = *{*g_1_, . . ., g_n_, ..., g_N_*}, a set of *M *biological samples *S = *{*s_1_, . . .,s_m_, ..., s_M_*}, and a series of *L *time points *T = *{*t_1_, . . ., t_l_, ..., t_L_*}, a 3D microarray gene expression dataset or gene-sample-time (GST) dataset is a real-values *N × M × L *matrix, *A = *{*a_nml_*}. Each entry *a_nml _*represents the expression level of gene *g_n _*in sample *s_m _*at time *t_l_*. We shall also refer to the 3D data as a set: *A = *{*G, S, T*}. In the sequel, we denote the expression level of gene *g_n _*in sample *s_m _*across the time points as *f_nm_*(*T*), which is a row vector. The expression profile of gene *g_n _*in all samples and across the time-series *f_n_*(*s,T*), which is a 2D matrix. Thus the 3D gene expression matrix can be viewed as a set of 2D matrices in the horizontal plane: Equation 1.

(1) $$A=\left[\begin{array}{c} f_{1}\left(s,T\right)\\ f_{2}\left(s,T\right)\\ \vdots \\ f_{n}\left(s,T\right)\\ \vdots \\ f_{N}\left(s,T\right) \end{array}\right]$$

A 3D cluster or tricluster is a 3D submatrix *C = *{*c_ijk_*} of *A*, or a subset *C = *{*I, J, K*}, with *I ⊆ G, J ⊆ S*, and *K ⊆ T*, such that the content of *C = *{*c_ijk_*} (*i*∈*I, j*∈*J*, and *k*∈*K*), verifies a desired pattern: constant, coherent values, or order preserving.

### Problem statement

Given the 3D matrix *A *as defined above and an ordering ranking threshold *δ*, find all triclusters *C = *{*I, J, K*}, with minimum number of genes *I_min_*, minimum number of samples *J_min_*, such that the content of each *C = *{*c_ijk_*} is order preserving over a given segment of a time-series. Since we are dealing with short time-series in this study, we set *K = T*. Thus we are interested in patterns that increase, decrease, or stay constant coherently across the entire time-series experiments. Note that constant patterns and coherent values are subsets of order preserving clusters.

### OPTricluster

Our triclustering algorithm identifies triclusters of genes with expression level having same direction across the time series experiments in subsets of samples. OPTricluster takes into consideration the sequential nature of the time-series and is able to cope with the effect of noise through the order preserving approach. Basically, for a given subset of samples, we say that a tricluster is order preserving if there exists a permutation of the time points such that the expression levels of the genes are monotonic functions. Such a matrix is defined by Equation 2 below. In this example, it is obvious that whatever permutations of its columns that we do, the variation between the values in its rows across the columns will always follow the same patterns.

(2) $$\left[\begin{array}{cccc} 2 & 8 & 5 & 3\\ 0 & 7 & 4 & 1\\ 1 & 5 & 3 & 2 \end{array}\right]$$

Furthermore, there exists a permutation of its columns (1, 4, 3, 2) or (2, 3, 4, 1) such that the values of its rows are monotonic increasing or decreasing function, respectively.

**Figure 12:**
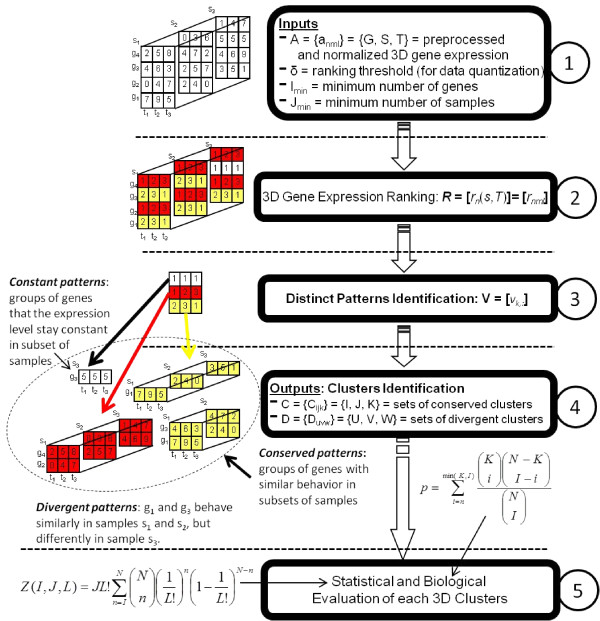
**OPTricluster illustration Example illustrating the different steps of the OPTricluster algorithm**.

In all, after the data pre-processing and normalization, OPTricluster has five main steps (Figure 12). First, OPTricluster performs the gene expression data quantization. Second, it ranks the expression level of the genes across the time-dimension in all the samples for a given *δ*. Third, it identifies the set of distinct coherent 3D patterns in the 3D dataset. Fourth, triclusters of coherent patterns are formed by assigning genes with similar ranking along the time-dimension and across subsets of samples to the same group, then divergent patterns are identified. Finally, statistical significance and biological evaluation of the triclusters identified are performed.

#### Gene expression matrix quantization

The first step of OPTricluster which is in fact optional consists of performing the gene expression data quantization. This is due to the fact that we are not only working with noisy data, but also DNA experimental data contains missing values. Many techniques are available in the literature to deal with noise through data quantization and to recover missing values by imputation [13]. OPTricluster uses the following approach for data quantization. Given the ordering and ranking threshold *δ*, for each row (gene) and a given sample, OPTricluster computes *E = *(*b_E _- b*_0_)/*δ*, where *b_0 _= min*(*f_nm_*(*T*)), *b_E _= max*(*f_nm_*(*T*)), and *δ ≠ 0*. Note that *E *is always rounded to the next integer of *(b_E _- b*_0_)/*δ*. Then the interval [*b*_0_, *b_E_*] is divided into *E *equal intervals: [*b*_0_, *b_E_*] *= *[*b*_0_, *b*_1_[U *..*.U [*b*_*e-*1_, *b_e _*[U ...U [*b*_*e-*1_, *b_E_*], where *b_e _= b*_0 _+ *eδ*, and *e = *1 to *E*. Finally, a new expression level of the corresponding gene in the considered sample at the given time point is obtained using Equation 3.

(3) $$\alpha_{e}=\left(b_{e}+b_{e-1}\right)/2$$

Specifically, if the expression level *f_nml _*of the corresponding gene *g_n _*in the given sample *s_m _*and at a given time points *t_l _*falls in the interval [*b*_*e-*1_, *b_e_*[, then it is quantized to the centroid *α_e _*of that interval. Subsequently, each centroid *α_e _*is assigned a ranking order (see below).

Note that, for a given microarray data, if the distribution of the expression levels of most genes in each sample and across the time points are densely located in a certain region, depending on the *δ *value, the quantization scheme defined above will likely put the densely related genes in the same cluster, usually the constant ones. Thus the value of *δ *should be chosen based on the following criteria: distribution of the gene expression data, the desired fold change between the expression levels of the genes across the time series, and based on the expected level of noise in the microarray data. Since the later is difficult to measure in real applications, one can also run the algorithm several times for a given value of *δ*, with perturbation values below and above it, and consider only the clusters that the content does not change a lot.

#### 3D rank expression matrix

OPTricluster then uses the quantized 3D gene expression matrix to generate a 3D rank expression matrix in the second step. A 3D rank expression matrix is an *N × M × L *matrix, *R = *[*r_n_*(*s,T*)] *= *[*r_nml_*], in which every row along the time-dimension for a given sample is a vector of the ranks of the corresponding expression values in *A*, in an increasing or decreasing order. For example, if the expression levels of gene *g_n _*in sample *s_m _*along the time-dimension is *f_nm_*(*T*) *= *[*1.5, 3, 0.5*] at *δ *≤1.0, then, the corresponding row in the rank matrix would be *r_nm_*(*T*) *= *[1-3] in the increasing order. The ranking matrix of the example of an order preserving matrix defined by Equation 2 above will be:

(4) $$\left[\begin{array}{cccc} 1 & 4 & 3 & 2\\ 1 & 4 & 3 & 2\\ 1 & 4 & 3 & 2 \end{array}\right]$$

Without lost of generalities, one can easily see from this example (Equation 4) that the rows of the ranking matrix of an order preserving matrix will always be identical. In fact, this property is exploited below for the identification of order preserving patterns from a 3D short time series gene expression matrix. Note that, if more than two entries have the same value, they are given the same ranking. For example [*0.5, 3, 0.5*] would be [*1, 2, 1*]. This approach allows the identification of constant patterns.

There are several advantages associated with this transformation. First, it avoids the use of greedy algorithms, probabilistic approaches, and exhaustive permutations along the time-dimension, and thus speeds up the computation time. Second, it is obvious that for any *k >*1 rows *f_nm_*(*T*) of similar ranking *r_nm_*(*T*), under any permutation of the time points, their order is always preserved (example in Equation 4). Thus they will always belong to the same coherent tricluster. Since the rank is conserved under any permutation along the time-dimension of the 3D gene expression matrix and given that we are dealing with multiple samples at the same time, the probability that a random pattern might be picked up in a cluster is very low.

#### Identification of distinct patterns

In its third step, OPTricluster identifies the set of distinct 3D coherent patterns that can be found in the 3D gene expression matrix *A*. Given the 3D gene expression matrix *A = *[*f_n_*(*s,T*)*] *as defined above, with a set of genes *G = *{*g_1_, . . ., g_n_, ..., g_N_*}, a set of biological samples *S = *{*s_1_, . . .,s_m_, ..., s_M_*} and a series of time points *T = *{*t_1_, . . ., t_l_, ..., t_L_*}, we define the biological sample space *Ω *as the set of all possible combination of the samples, and *Γ *their number. That is, if *S = *{*s_1 _s_2 _s_3_*} as in Figure 12, then the biological sample space would be *Ω = *{{*s_1_, s_2_, s_3_*}, {*s_1_, s_2_*}, {*s_1_, s_3_*}, {*s_2_, s_3_*}, {*s_1_*}, {*s_2_*}, {*s_3_*}} and *Γ = 7*. Recall that *r_n_(s,T) *corresponds to a row vector of the ranks of the nth gene, in sample *s *and across the entire time series *T*. Hence, for each combination *Ω_i_*∈*Ω*, the exact number *h_i _*of distinct order preserving triclusters that can be found in the 3D dataset is the number of distinct 2D *r_n_*(*Ω_i_,T*) matrices of its corresponding 3D ranked matrix *R*. Thus, the set of 3D distinct order preserving patterns, *V*, can be identified by considering *R *as a set of 2D matrices *r_n_*(*Ω_i_,T*), that is*, R = *{*r_1_*(*Ω_i_,T*)*, r_2_*(*Ω_i_,T*)*, ..., r_n_*(*Ω_i_,T*)*, ..., r_N_*(*Ω_i_,T*)}, and identify all distinct *r_n_*(*Ω_i_,T*) in it. From the above definitions, one can easily show that the exact number *Λ *of order preserving triclusters in the 3D gene expression matrix is:

(5) $$\Lambda =\overset{\Gamma }{\underset{i=1}{\sum }}h_{i}$$

Recall that *h_i _*is the number of distinct 2D *r_n_*(*Ω_i_,T*) (rank matrices)corresponding to each *Ω_i_*∈*Ω *as defined above.

#### Conserved clusters identification

Once the exact number of distinct 3D order preserving patterns has been identified, for each *Ω_i_*∈*Ω*, OPTricluster assigns each gene to one of the *h_i _*groups by comparing each distinct pattern *v_k _*of *V *(*V*: set of 3D distinct order preserving patterns identified from the previous step) to *r_n_*(*Ω_i_,T*), and assign gene *g_n _*to the order preserving tricluster *C*{*k*} each time *r_n_*(*Ω_i_,T*) = *v_k _*. This approach is guaranteed to identify all order preserving triclusters of size *I × J × K*, with *I_min _≤ I ≤ N*, *J_min _≤ J ≤ M*, and *K = L*, where *I_min _*and *J_min _*are the minimum number of genes and samples in a tricluster, respectively.

Since the goal of the OPTricluster algorithm is to study the similarities and differences between samples in terms of the expression profile of all genes, I_min _and J_min _should be set to 1. In this case, the algorithm will identify all the conserved clusters and perform comparison between them at the single sample and single gene level in the divergent patterns identification step, as explained below.

#### Divergent patterns identification

The 3D procedure as presented above identifies sets of genes that behave similarly (same OP patterns) across the subsets of samples considered. The sets of divergent patterns *D *can be easily derived from the sets of conserved ones using Equation 5.

(6) $$D_{pq}=C\left\{p\right\}-C\left\{q\right\}=\left\{\begin{array}{c} I_{p}-I_{q}\\ J_{p}-J_{q} \end{array}\right),\;\;p\neq q$$

*C*{*p*} *= *{*I_p_, J_p_, K_p_*} and *C*{*q*} *= *{*I_q_, J_q_, K_q_*} are two conserved triclusters (similar OP patterns). Basically, Equation 5 identifies sets of genes that are co-expressed in the subset of sample in *C*{*p*}, but split and co-expressed differently in one or more samples in *C*{*q*}. For example, if clusters *C*{*p*} and *C*{*q*} have the same OP patterns, and if *C*{*p*} *= *{{*g_1_, g_2_, g_3_, g_4_*}, {*s_1_, s_2_*}, {*t_1_, t_2_, t_3_*}} and *C*{*q*} *= *{{*g_1_, g_2_*}, {*s_1_, s_2_, s_3_*}, {*t_1_, t_2_, t_3_*}}, then *D_pq _= *{{*g_3_, g_4_*}, {*s_3_*}, {*t_1_, t_2_, t_3_*}}, meaning that genes {*g_3_*,*g_4_*} have different behaviour in {*s_3_*} compared to {*s_1_*,*s_2_*}. The computational burden of this step is reduced because only triclusters with same OP or ranking patterns are compared. This is due to the fact that ranking patterns are associated with the expression profile and are unique to each cluster for a given subset of samples.

### Statistical significance and complexity analysis

The statistical significance of each identified tricluster with *I *genes and *J *samples is assessed by computing the tail probability that a random dataset of size *N × M × L *will contain an order preserving tricluster with *I *or more genes and *J *or more samples in it. In principle, the probabilistic description of the reference *3D *random matrix would be that of the observed noise in the microarray experiment [5,14,33]. Since this distribution is difficult to calculate in closed form, the upper bound of this tail probability is estimated using the same approach as in [5,13,14,33]. Assuming that we have a dataset with *L *time points that are independent and identically distributed according to the uniform distribution, the probability that a random gene-sample supports a given cluster is equal to the number of possible time points permutations or *1/L!*. Since the genes and samples are assumed to be independent, the probability of having at least *I *genes and *J *samples in the cluster is the *I *-tail of the (*N,(1/L!)) *binomial distribution, i.e.:

(7) $$P\left(X\geq I\right)=\overset{N}{\underset{n=I}{\sum }}\left(\begin{array}{c} N\\ n \end{array}\right){\left(\frac{1}{L!}\right)}^{n}{\left(1-\frac{1}{L!}\right)}^{N-n}$$

As there are *Ls *= *L! *ways to choose an OP tricluster of size *L*, the following expression *Z(I, J, L) *is an upper bound on the probability of having a tricluster of size *L *with *I *or more genes and *J *samples:

(8) $$Z\left(I,J,L\right)=JL!\overset{N}{\underset{n=I}{\sum }}\left(\begin{array}{c} N\\ n \end{array}\right){\left(\frac{1}{L!}\right)}^{n}{\left(1-\frac{1}{L!}\right)}^{N-n}$$

We use this bound to estimate the significance of any given tricluster of size *L *with *I *genes and *J *samples. The best tricluster is the one with the largest statistical significance, i.e., the one with the *smallest Z(I, J, L) *Therefore, as long as that upper bound probability is smaller than any desired significance level, the identified tricluster in the real 3D gene expression matrix will be statistically significant.

The overall complexity of the triclustering algorithm is *O*(*NΓΛ*). Recall that the 3D short time-series gene expression data *A *is an *N × M × L *matrix. The 3D rank matrix can be identified within *O*(*NML*). The set of distinct 3D patterns can be identified with *O*(*NΓ*). Finally, the set of coherent conserved triclusters can be identified within *O*(*NΓΛ*). In all, the complexity of the triclustering algorithm is *O*(*NML*) *+ O*(*NΓ*) *+ O*(*NΓΛ*), which is *O*(*N*(*ML + Γ + ΓΛ*)). Note that the complexity for identifying the sets of divergent patterns from convergent ones is negligible. Since *ΓΛ *>*Γ *and *ΓΛ *>*ML*, the overall time complexity is *O*(*NΓΛ*).

## Competing interests

The authors declare that they have no competing interests.

## Authors' contributions

Designed the algorithm and performed clustering: ABT. Implemented the algorithm in Matlab and Java: ABT. Conceived and designed experiments: ABT, SP, and YP. Performed the *Arabidopsis *and *Brassica *biological experiments and contributed the data: HS, PF, YH, JZ, DH, and AC. Analyzed the data: ABT, SP, ZL, and YP. Wrote the first draft manuscript: ABT. Commented on the manuscript with important intellectual contributions: SP, FF, PF, JZ, ZL, AC and YP. Revised the manuscript: ABT and YP. (All authors read and approved the final manuscript.)

## Supplementary Material

Additional file 1 **OPTricluster Java package**. Click here for file

Additional file 2 **OPTricluster user manual**. Click here for file

Additional file 3 **Expression profile of NPR1 in different samples The axis corresponds to the time point experiments, the y-axis the expression level in Log2. Each curve corresponds to a sample**. Click here for file

Additional file 4 **Statistic of differences between inner and outer cotyledons The x-axis corresponds to the combination of time points, the y-axis the number of genes**. Click here for file

Additional file 5 Gene Ontology analysis of whole seed *Brassica napus *clusters GO analysis of the 11 clusters in whole seed development *Brassica napus*. Click here for file
