# Supplementary material for: Mining biological information from 3D short time-series gene expression data: the OPTricluster algorithm
Source: BMC Bioinformatics. 2012 Apr 4;13:54. doi: 10.1186/1471-2105-13-54 (PMC3376030; doi:10.1186/1471-2105-13-54)
Supplement: Additional file 1 — OPTricluster Java package. [file 1471-2105-13-54-S1.ZIP › OPTricluster/Manual/OPTricluster_Quick_Tutorial.pdf]

OPTricluster Input Interface

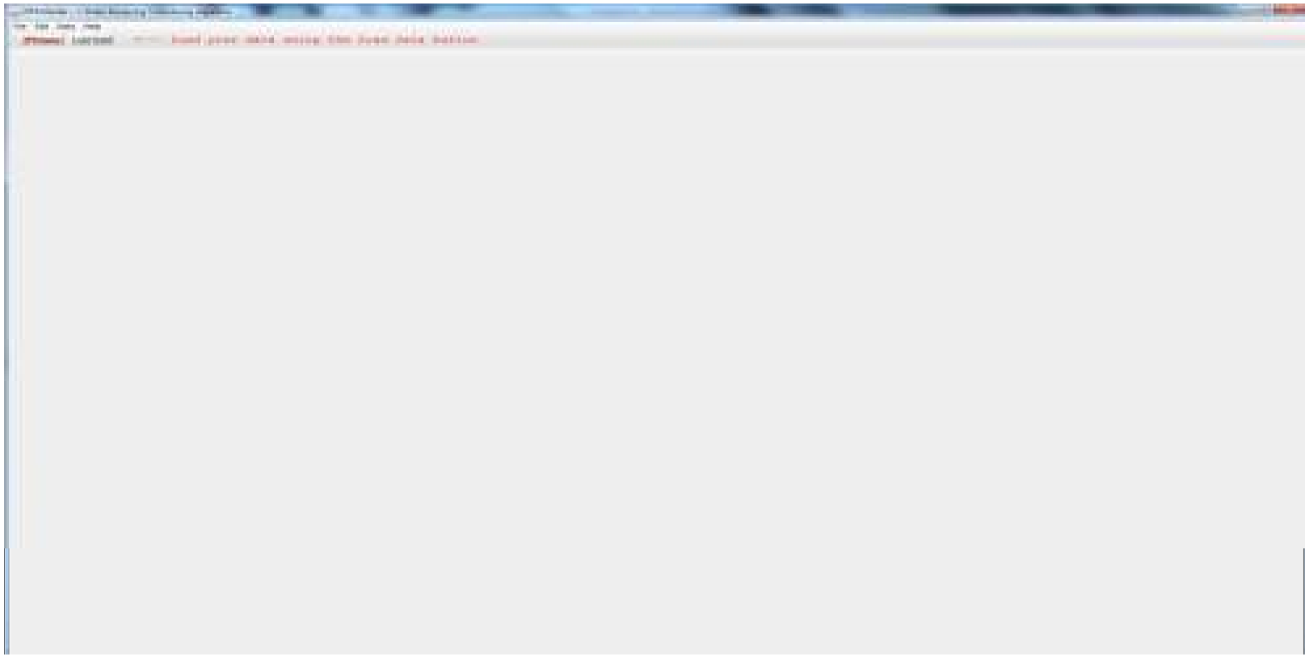

To quickly test OPTricluster, 2 datasets are included in the software. They can be accessed via the Data menu. (**Data → Testing)**

- Plasmodium dataset used (Cernetichet al., 2006). This dataset is used in this study.

To obtain the same results, use the following parameters

- minimum number of gene = 1
- minimum number of sample = 1
- ranking threshold = 0.31

•The Arabidopsis dataset (Matsui et al., 2008)

- minimum number of gene = 1
- minimum number of sample = 1
- ranking threshold = 1

To load your own dataset (**Data → New**, or **Load Data** button) respect the data file convention.

Sample input data file

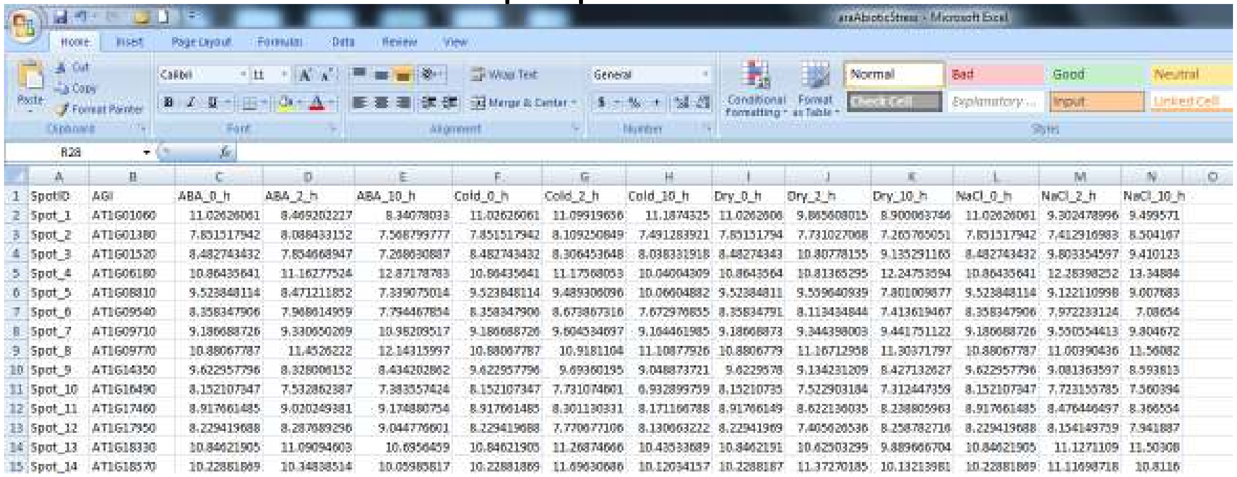

	A	B	C	D	E	F	G	H	I	J	K	L	M	N
1	SpotID	AGI	ABA_0_h	ABA_2_h	ABA_10_h	Cold_0_h	Cold_2_h	Cold_10_h	Dry_0_h	Dry_2_h	Dry_10_h	NaCl_0_h	NaCl_2_h	NaCl_10_h
2	Spot_1	AT1G01060	11.02626061	8.469202227	8.34078033	11.02626061	11.09919656	11.1874325	11.0262606	9.865608015	8.900063746	11.02626061	9.302478996	9.499571
3	Spot_2	AT1G01380	7.851517942	8.088433152	7.568799777	7.851517942	8.109250849	7.491283921	7.85151794	7.731027068	7.265765051	7.851517942	7.412916983	8.504167
4	Spot_3	AT1G01520	8.482743432	7.854668947	7.268830887	8.482743432	8.306453648	8.038331918	8.48274343	10.80778155	9.135291165	8.482743432	9.803354597	9.410123
5	Spot_4	AT1G06180	10.86435641	11.16277524	12.87178783	10.86435641	11.17568053	10.04004309	10.8643564	10.81365295	12.24753594	10.86435641	12.28398252	13.34884
6	Spot_5	AT1G08810	9.523848114	8.471211852	7.339075014	9.523848114	9.489306096	10.06604882	9.52384811	9.559640939	7.801009877	9.523848114	9.122110998	9.007683
7	Spot_6	AT1G09540	8.358347906	7.968614959	7.794467854	8.358347906	8.673867316	7.672976855	8.35834791	8.113434844	7.413619467	8.358347906	7.972233124	7.08654
8	Spot_7	AT1G09710	9.186688726	9.330650269	10.98209517	9.186688726	9.604534697	9.164461985	9.18668873	9.344398003	9.441751122	9.186688726	9.550554413	9.804672
9	Spot_8	AT1G09770	10.88067787	11.4526222	12.14315997	10.88067787	10.9181104	11.10877926	10.8806779	11.16712958	11.30371797	10.88067787	11.00390436	11.56082
10	Spot_9	AT1G14350	9.622957796	8.328006152	8.434202862	9.622957796	9.69360195	9.048873721	9.6229578	9.134231209	8.427132627	9.622957796	9.081363597	8.593813
11	Spot_10	AT1G16490	8.152107347	7.532862387	7.383357424	8.152107347	7.731074601	6.932899759	8.15210735	7.522903184	7.312447359	8.152107347	7.723155785	7.560394
12	Spot_11	AT1G17460	8.917661485	9.020249381	9.174880754	8.917661485	8.301130331	8.171166788	8.91766149	8.622136035	8.238805963	8.917661485	8.476446497	8.366554
13	Spot_12	AT1G17950	8.229419688	8.287689296	9.044776601	8.229419688	7.770677106	8.130663222	8.22941969	7.405626536	8.258782716	8.229419688	8.154149759	7.941887
14	Spot_13	AT1G18330	10.84621905	11.09094603	10.6956459	10.84621905	11.26874666	10.42533689	10.8462191	10.62503299	9.889666704	10.84621905	11.1271109	11.50308
15	Spot_14	AT1G18570	10.22881869	10.34838514	10.05985817	10.22881869	11.69630686	10.12034157	10.2288187	11.37270185	10.13213981	10.22881869	11.11698718	10.8116

- First column(SpotID) is optional
- Second column Gene Name (this column becomes the first column if no SpotID).
- Respect the first row convention. Sample_Time_Unit
- Save the file as Tab Delimited File.

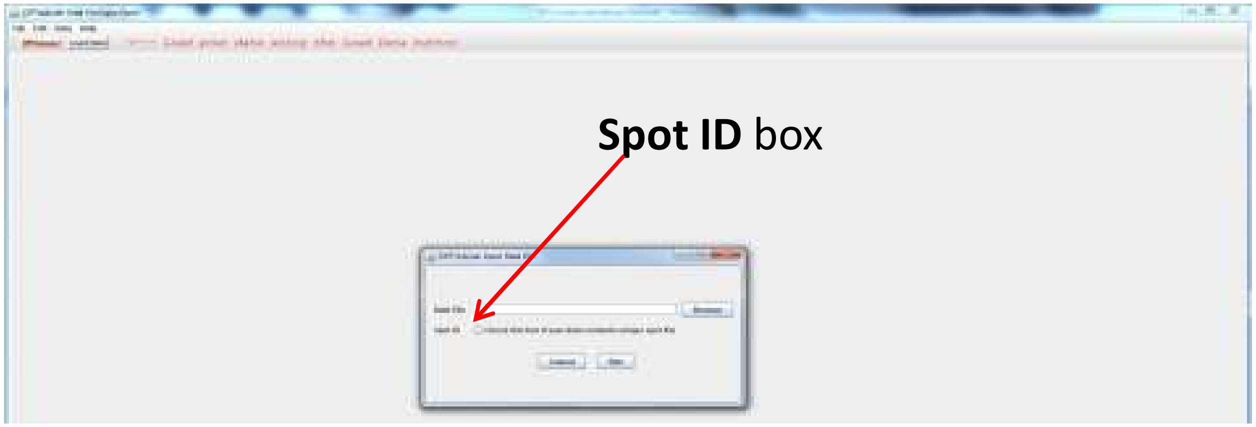

When SpotID is included in the data file, make sure you check the Spot ID box located on the OPTricluster **Input Data File**.

Once the data is loaded, your OPTricluster interface should look like this:

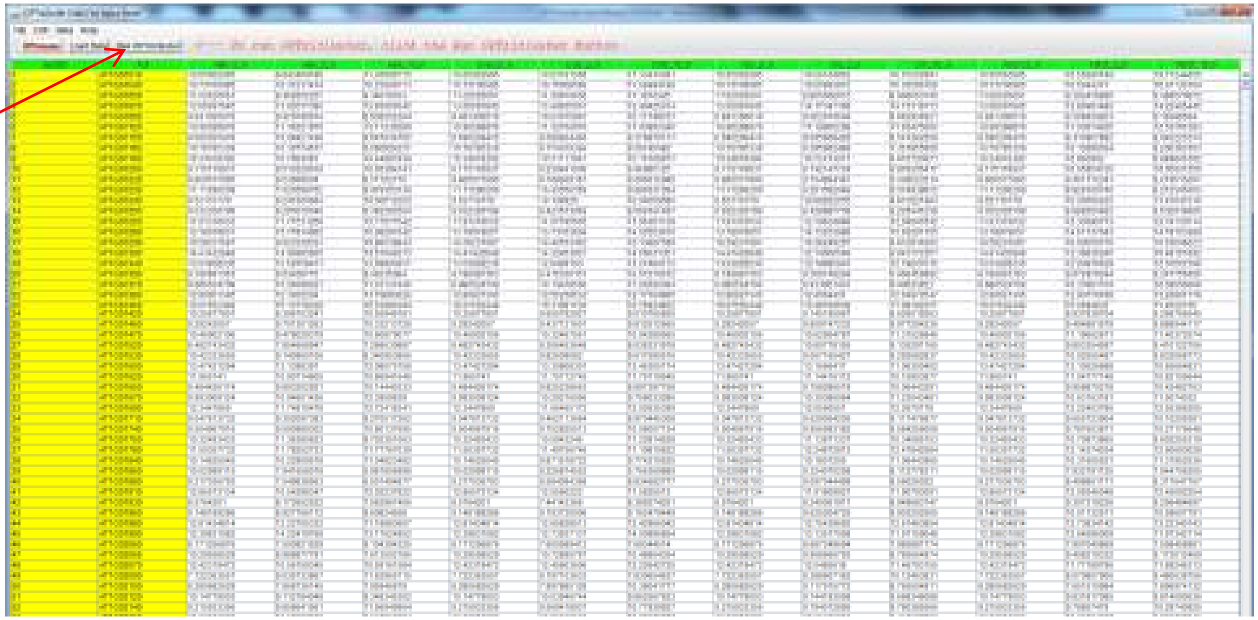

Click the Run OPtricluster button
to call the **OPTricluster Input Parameters**

Input the parameters and
click run

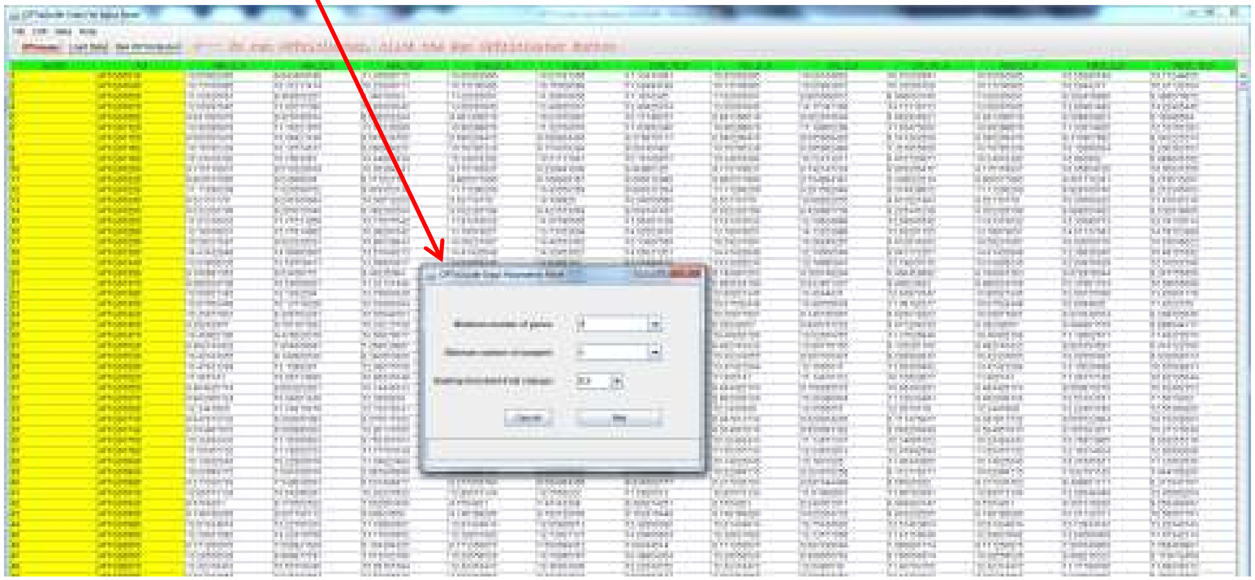

Your OPTricluster Interface should look like this once you have clicked on run .

Use the **Select Patterns** Drop down menu to explore Conserved, constant, and divergent patterns

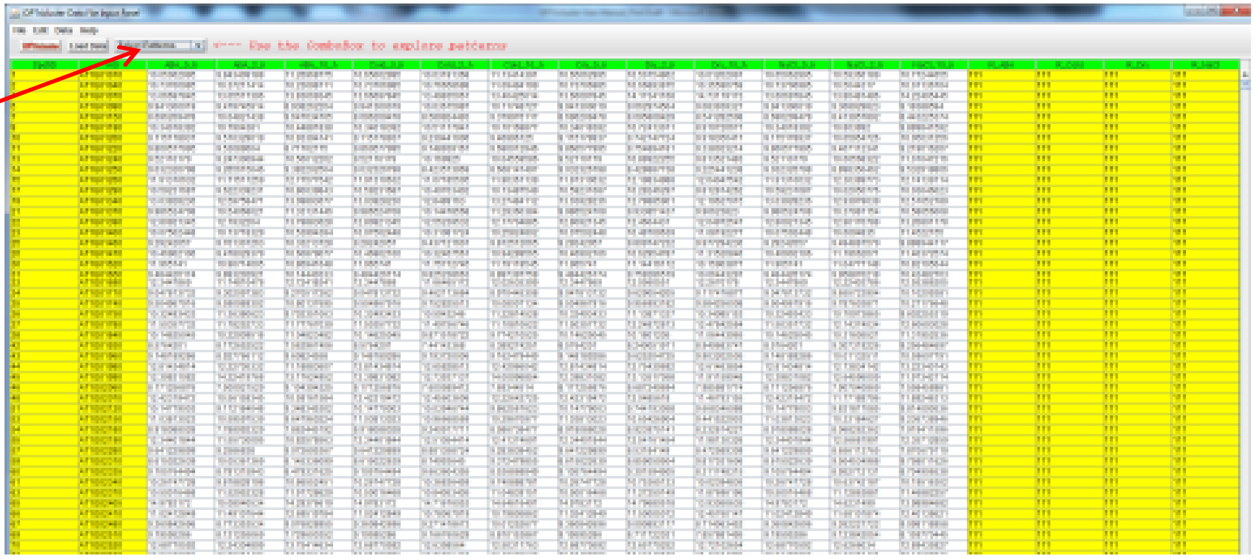

If you selected Conserved Patterns for example, your OPTricluster Interface should look like this once you have clicked on run .

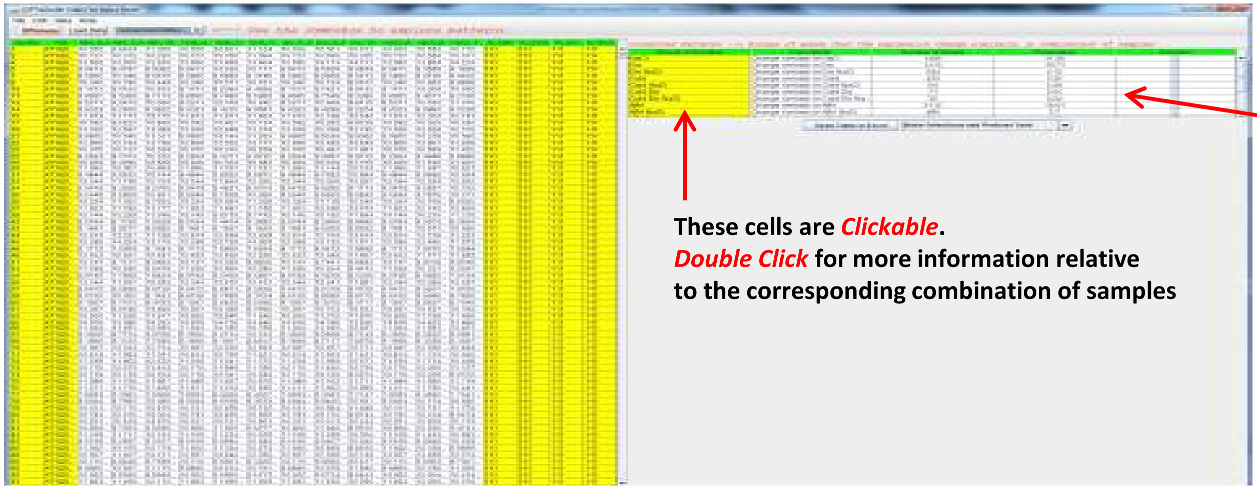

Sample Table

These cells are *Clickable*.
***Double Click* for more information relative to the corresponding combination of samples**

Your OPTricluster interface should look like this if you had double clicked in a subset of samples cell (Sample Table).

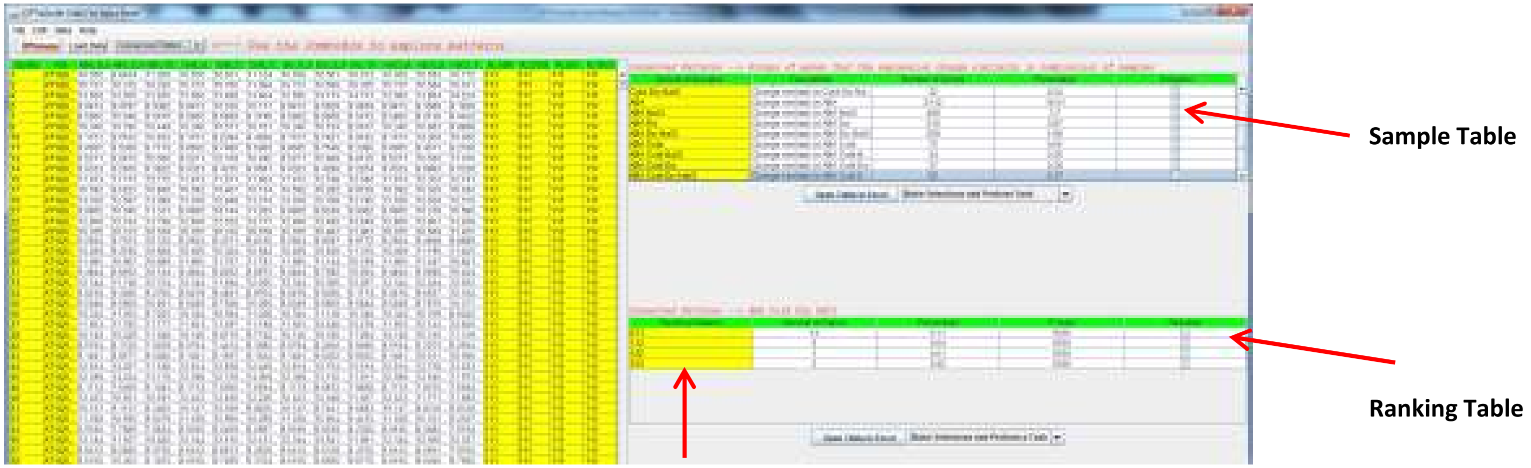

These cells are *Clickable*.
***Double Click* for more information relative to the corresponding ranking**

Your OPTricluster interface should look like this if you had double clicked in a **Ranking Patterns** (Ranking Table).

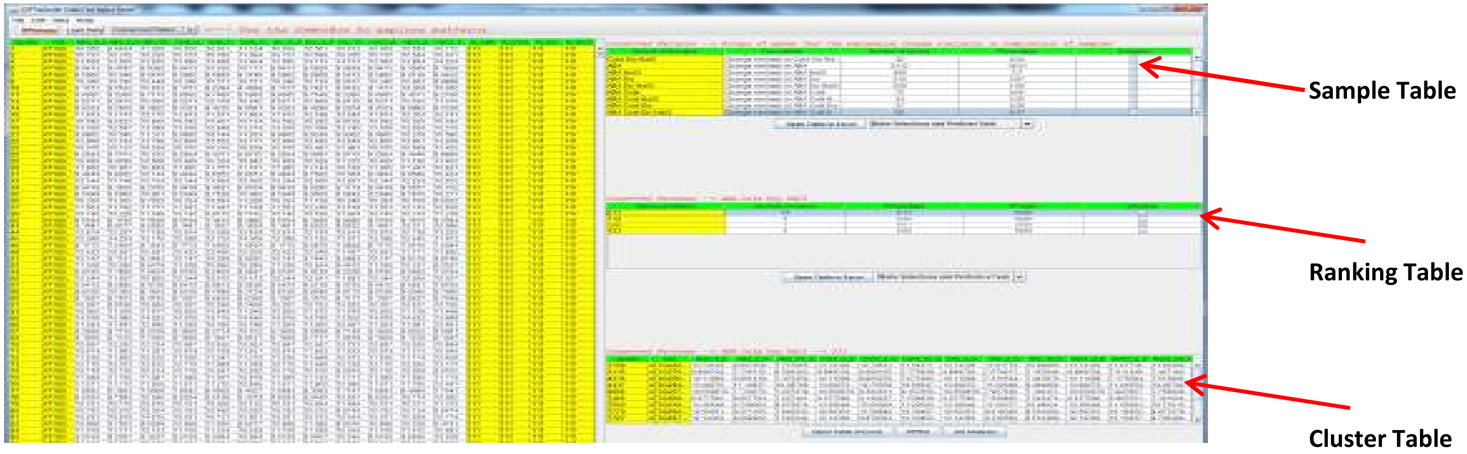

Use the **XYPlot** button to view the gene expression plot of the corresponding cluster
Use the **GO Analysis** button for Gene Ontology analysis
Use the **Open Table in Excel** Button to export your data into Excel

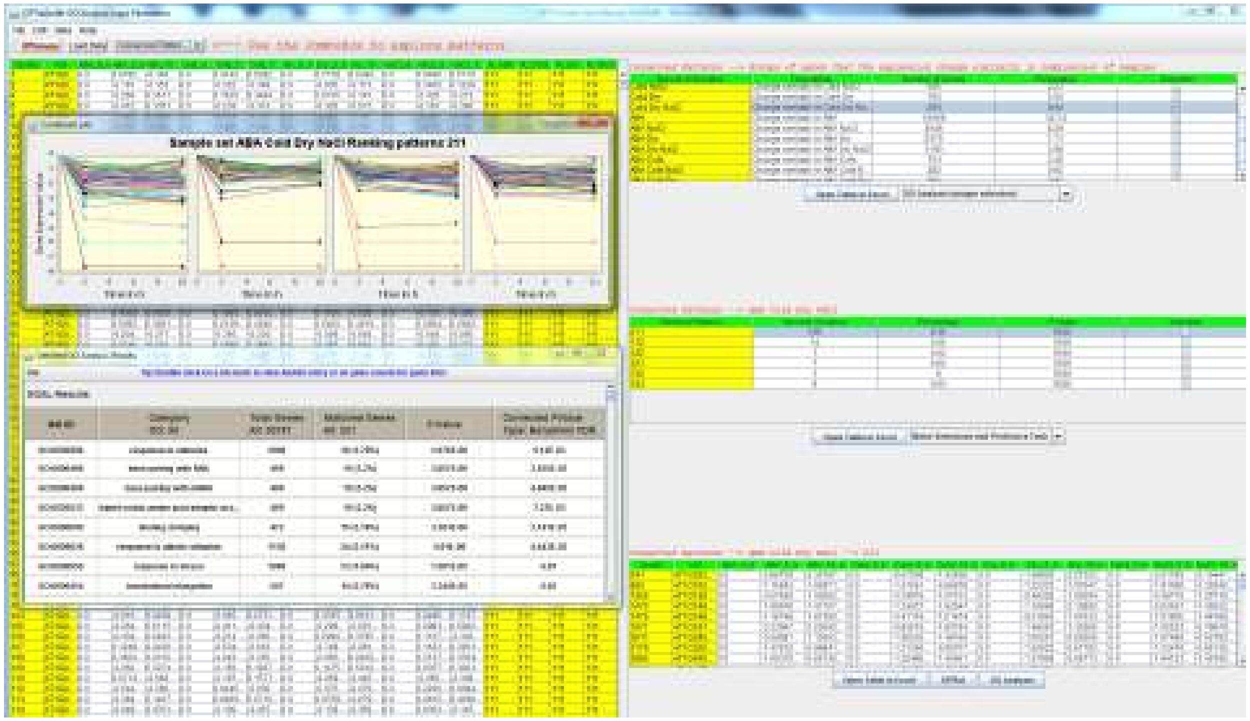

For other functionalities of OPTricluster, refer to the OPTricluster User Manual
Help→ User Manual

Enjoy!!!!
