# Supplementary material for: Mining biological information from 3D short time-series gene expression data: the OPTricluster algorithm
Source: BMC Bioinformatics. 2012 Apr 4;13:54. doi: 10.1186/1471-2105-13-54 (PMC3376030; doi:10.1186/1471-2105-13-54)
Supplement: Additional file 1 — OPTricluster Java package. [file 1471-2105-13-54-S1.ZIP › OPTricluster/Manual/OPTricluster_User_Manual.pdf]

Order Preserving Triclustering Algorithm

User Manual

(Version1.0)

Alain B. Tchagang alain.tchagang@nrc-cnrc.gc.ca
Fazel Famili fazel.famili@nrc-cnrc.gc.ca
Sieu Phan sieu.phan@nrc-cnrc.gc.ca
Ziying Liu ziying.liu@nrc-cnrc.gc.ca

Knowledge Discovery Group,
Institute for Information Technology
National Research Council Canada
1200 Montreal Road, Ottawa, ON K1A 0R6, Canada

© 2012

Content

I. Introduction **2**
- I.1. OPTricluster clustering method overview **2**
- I.2. Citing OPTricluster **2**
- I.3. Manual overview **2**

II. Running OPTricluster **3**

III. Input Interface **3**
- III.1. Menu bar **4**
- III.2. Tool bar **4**
- III.3. Working space **4**

IV. Data Analysis with OPTricluster **5**
- IV.1. Expression data info **5**
- IV.2. OPTricluster input parameters interface **7**
- IV.3. Exploring OPTricluster patterns **8**
  - i. Conserved patterns **9**
  - ii. Divergent patterns **13**
  - iii. Constant patterns **13**

V. Integration with Gene Ontology **14**

VI. Integration with JFreeChart **15**

VII. References **16**

I. Introduction

OPTricluster stands for Order Preserving Triclustering Algorithm, a software package designed for clustering, visualizing, and studying similarities and differences between samples in terms of temporal expression profiles in 3D short time series gene expression data (2-4 samples, 3-8 time points) from microarray experiments [1]. OPTricluster implements a novel method for analyzing and visualizing 3D short time series expression data using the order preserving concept on the time dimension and a combinatorial approach on the sample dimension. OPTricluster is integrated with the Gene Ontology (GO) [2-3] allowing efficient biological interpretations of the data. It is also integrated with the JFreeChart library [4].

I.1. OPTricluster clustering method overview

The triclustering algorithm we developed identifies triclusters of genes with expression level having same direction across the time point experiments in subsets of samples. OPTricluster takes into consideration the sequential nature of the time-series and is able to cope with the effect of noise through the order preserving approach. Basically, for a given subset of samples, we say that a tricluster is order preserving if there exists a permutation of the time points such that the expression levels of the genes are monotonic functions. In all, after the data pre-processing and normalization, OPTricluster has five main steps. First, OPTricluster performs the gene expression data quantization. Second, it ranks the expression level of the genes across the time-dimension in all the samples for a given filtering threshold (δ). Third, it identifies the set of distinct coherent 3D patterns in the 3D dataset. Fourth, triclusters of coherent patterns are formed by assigning genes with similar ranking along the time-dimension and across subsets of samples to the same group, then divergent patterns are identified. Finally, statistical significance and biological evaluation of the triclusters identified are performed. For more details about OPTricluster methodology, see [1].

I.2. Citing OPTricluster

To cite the OPTricluster software please references the paper:

Tchagang A.B, Phan S, Famili F, Shearer H, Fobert P, Huang Y, Zou J, Huang D, Cutler A, Liu Z, and Pan Y. Mining biological information from 3D short time-series gene expression data: the OPTricluster algorithm. *BMC Bioinformatics, 2012,* ***under review***.

I.3. Manual overview

The remainder of the main portion of the manual contains five sections. Section 2 contains instructions on installing and starting OPTricluster. Section 3 discusses the input to OPTricluster. Section 4 describes data analysis scenarios using OPTricluster, which allows users to explore and

visualize different type of patterns. Section 5 describes the integration of OPTricluster with Gene Ontology, and section 6 its integration with the JFreeChart library.

II. Running OPTricluster

- To use OPTricluster a version of Java 1.6 or later must be installed. If Java 1.6 or later is not currently installed, then it can be downloaded from http://www.java.com.
- To install OPTricluster simply save the file OPTricluster.zip locally and then unzip it. This will create a directory called OPTricluster.
- To execute OPTricluster in Windows with its default initialization options simply double click on the file runOPTricluster_Windows in the OPTricluster directory.
- To execute OPTricluster in Linux with its default initialization options simply double click on the file runOPTricluster_Linux in the OPTricluster directory.
- To execute OPTricluster from a command line, change to the OPTricluster directory then type: java -mx1024M -jar OPT.jar.
- By only double clicking on the OPT.jar file in the OPTricluster directory, or type java OPT.jar in the command line, OPTricluster will run without its defaults initialization options.

III. Input Interface

The first window that appears after OPTricluster is launched is the user input interface (**Figure 1**), which includes three sections: the *menu bar*, the *tool bar*, and the *working space*.

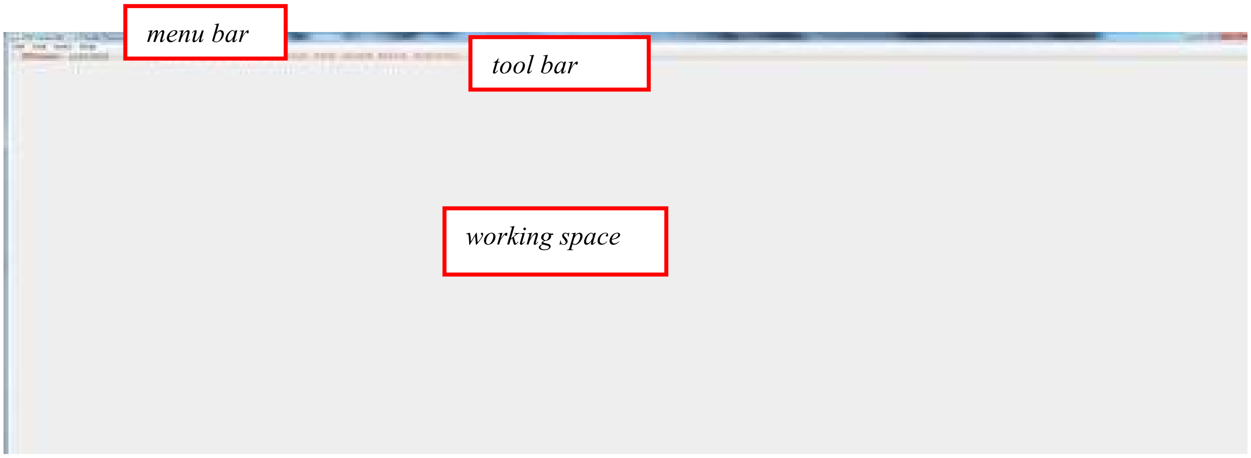

Figure 1: Main user input interface of OPTricluster software. It is the first screen that appears when OPTricluster is launched. It is divided into three sections: the *menu bar*, the *tool bar*, and the *working space*.

III.1. *tool bar*

The *tool bar* (**Table 1**) contains several command buttons which in some cases are short-cuts to the menu items of the *menu bar*.

Table 1: Description of the OPTricluster *tool bar*

**OPTricluster *tool bar***	**Functions**
OPTricluster	Information relative to the current version of OPTricluster
Load Data	Load new data for analysis
Run OPTricluster	Called the OPTricluster input parameters panel
Select Patterns	Allow user to select type of patterns to explore (Conserved, Divergent, Constant)
Label	Tell the user what to do at each step of the analysis

III.2. *menu bar*

The *menu bar* (**Table 2**) contains four menus; it can be used to access the functionalities of OPTricluster.

Table 2: Description of the OPTricluster *menu bar*

**OPTricluster *menu bar***	**Menu items**	**Functions**
File	New	Open a new OPTricluster window while keeping the last one opened
	Refresh	Refresh the current OPTricluster window
	Close	Close the current OPTricluster window
	Exit	Exit OPTricluster (close all the opened OPTricluster windows)
Edit	Open Data with Excel	Open the table data in excel
	Histogram	Distribution of the input data
Data	New	Allow the user to load new dataset for analysis
	Testing	Load datasets that can be used to test OPTricluster
	Update	Allow the user to update the Gene Ontology and the species annotation files
Help	About OPTricluster	Information relative to the current version of OPTricluster
	Licensing	Information relative to the license of OPTricluster
	Quick Tutorial	Quick tutorial in PDF format
	User Manual	User manual in PDF format

III.3. *working space*

The *working space* is reserved for displaying the results at each step of the analysis in the form of tables.

IV. Data Analysis with OPTricluster

IV.1. Expression data info

Once the OPTricluster is launched, the OPTricluster input interface appears (**Figure 1** above). From this screen a user specifies the input data file using the **Data → New** from the *menu bar* or the **Load Data** from the *tool bar*.

An input data file for OPTricluster is a tab delimited text file, which consists of gene symbols, time series expression values, and optionally spot IDs. Spot IDs uniquely identify an entry in the data file, and if they are not included in the data file, then they will be automatically generated. While spot IDs must be unique, the same gene symbol may appear multiple times in the data file corresponding to the same gene appearing on multiple spots on the array.

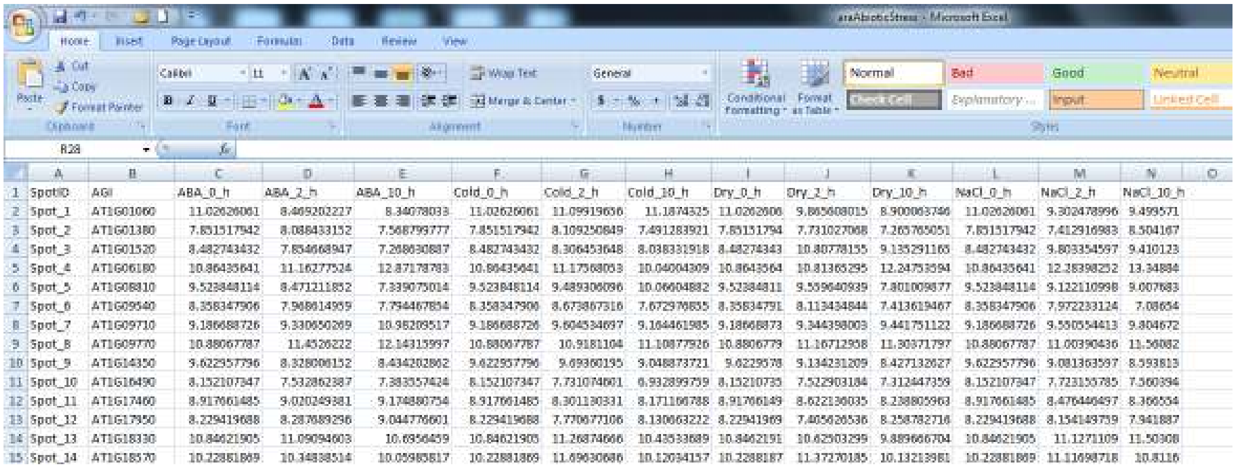

SpotID	AGI	ABA_0_h	ABA_2_h	ABA_10_h	Cold_0_h	Cold_2_h	Cold_10_h	Dry_0_h	Dry_2_h	Dry_10_h	NaCl_0_h	NaCl_2_h	NaCl_10_h
Spot_1	AT1G01060	11.02626061	8.469202227	8.34078033	11.02626061	11.09919656	11.1874325	11.0262606	9.865608015	8.900063746	11.02626061	9.302478996	9.499571
Spot_2	AT1G01380	7.851517942	8.088433152	7.568799777	7.851517942	8.109250849	7.491283921	7.85151794	7.731027068	7.265765051	7.851517942	7.412916983	8.504167
Spot_3	AT1G01520	8.482743432	7.854668947	7.268630887	8.482743432	8.306453648	8.038331918	8.48274343	10.80778155	9.135291165	8.482743432	9.803354597	9.410123
Spot_4	AT1G06180	10.86435641	11.16277524	12.87178783	10.86435641	11.17568053	10.04004309	10.8643564	10.81365295	12.24753594	10.86435641	12.28398252	13.34884
Spot_5	AT1G08810	9.523848114	8.471211852	7.339075014	9.523848114	9.489306096	10.06604882	9.52384811	9.559640939	7.801009877	9.523848114	9.122110998	9.007683
Spot_6	AT1G09540	8.358347906	7.968614959	7.794467854	8.358347906	8.673867316	7.672976855	8.35834791	8.113434844	7.413619467	8.358347906	7.972233124	7.08654
Spot_7	AT1G09710	9.186688726	9.330650269	10.98209517	9.186688726	9.604534697	9.164461985	9.18668873	9.344398003	9.441751122	9.186688726	9.550554413	9.804672
Spot_8	AT1G09770	10.88067787	11.4526222	12.14315997	10.88067787	10.9181104	11.10877926	10.8806779	11.16712958	11.30371797	10.88067787	11.00390436	11.56082
Spot_9	AT1G14350	9.622957796	8.328006152	8.434202862	9.622957796	9.69360195	9.048873721	9.6229578	9.134231209	8.427132627	9.622957796	9.081363597	8.593813
Spot_10	AT1G16490	8.152107347	7.532862387	7.383557424	8.152107347	7.731074601	6.932899759	8.15210735	7.522903184	7.312447359	8.152107347	7.723155785	7.560394
Spot_11	AT1G17460	8.917661485	9.020249381	9.174880754	8.917661485	8.301130331	8.171166788	8.91766149	8.622136035	8.238805963	8.917661485	8.476446497	8.366554
Spot_12	AT1G17950	8.229419688	8.287689296	9.044776601	8.229419688	7.770677106	8.130663222	8.22941969	7.405626536	8.258782716	8.229419688	8.154149759	7.941887
Spot_13	AT1G18330	10.84621905	11.09094603	10.6956459	10.84621905	11.26874666	10.43533689	10.8462191	10.62503299	9.889666704	10.84621905	11.1271109	11.50308
Spot_14	AT1G18570	10.22881869	10.34838514	10.05985817	10.22881869	11.69630686	10.12034157	10.2288187	11.37270185	10.13213981	10.22881869	11.11698718	10.8116

Figure 2: Above is a sample input data file (3D time series gene expression data) when viewed in Microsoft Excel. The first column SpotID is optional. When included, the SpotID box located on the OPTricluster input data file must be checked.

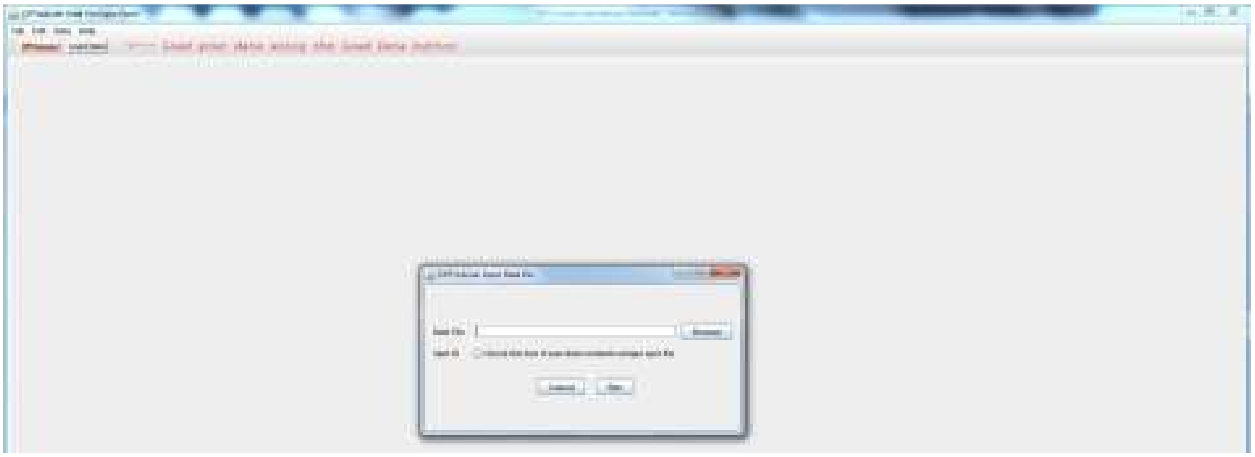

Figure 3: OPTricluster input interface showing the OPTricluster input data file when **Data → New** or **Load Data** is selected. The Spot ID box must be check if the data contains a SpotID column (**Figure 2**).

A sample data file representing a 3D time series gene expression data as it would appear in Microsoft Excel is shown in **Figure 2**. The first column is optional, and if included contains spot IDs. If the data file includes the spot IDs column, then the field Spot ID in the OPTricluster input Data File must be checked (**Figure 3**), otherwise the field must be unchecked.

The next column, or the first column if spot IDs are not included in the data file, contains gene symbols. If a gene symbol is not available then the field should not be left empty. A "*no_match*" can be placed in it. Both the spot ID field and the gene symbol field may contain multiple entries delimited by an underscore ("_").

The remaining columns contain the expression values in each sample and at each time point ordered sequentially based on time. If the data content missing values, they should be taken care of prior loading the data into OPTricluster. No field should be left empty.

The first row of the data file contains column headers, and each row below the column header corresponds to a spot on the microarray. The column header describes the sample, the time points and the unit of the time point and should respect the following format:

Sample_Time_Unit.

OPTricluster currently only accepts tab-delimited data file as input. A tab-delimited text file can easily be generated in Microsoft Excel by choosing Text (Tab delimited) as the Save as type under the Save As menu. Once the user selects the data file, it is loaded into the working space of OPTricluster **Figure 4**.

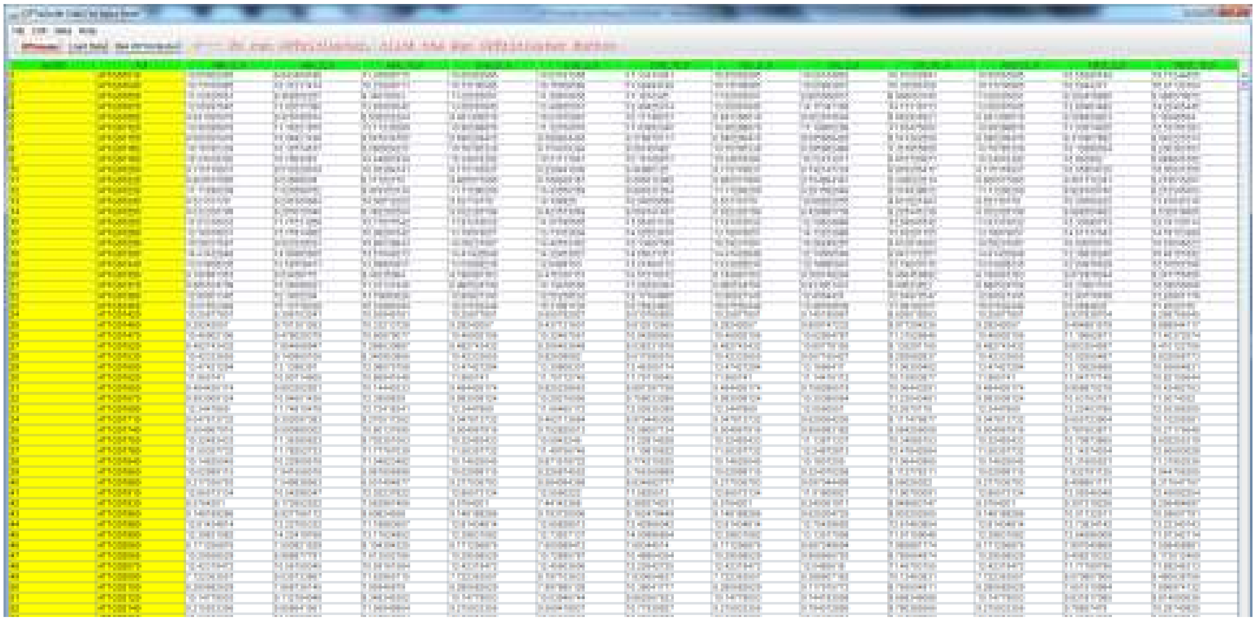

Figure 4: Example of the OPTricluster interface once the gene expression data is loaded.

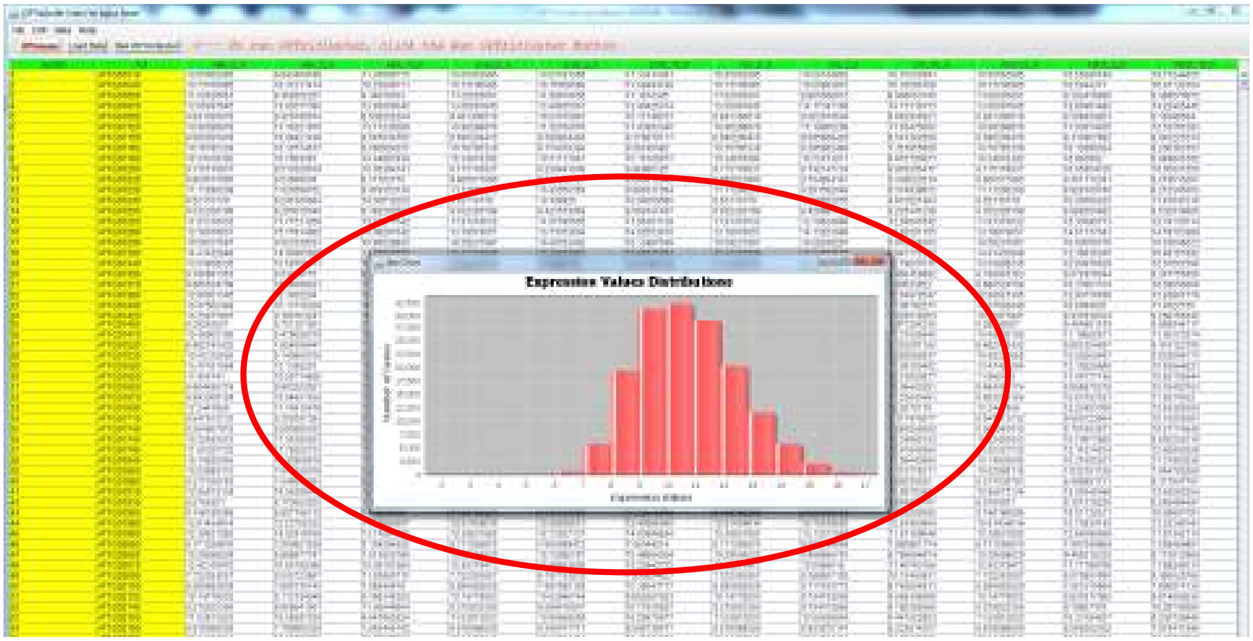

Figure 5: Example of the OPTricluster interface once the gene expression data is loaded and the user selects **Edit →Histogram** to view the distribution of the data.

IV.2. OPTricluster input parameters interface

Once the data is loaded, the user clicks on the **Run OPTricluster** from the *tool bar*. This action calls the OPTricluster input parameters interface (**Figure 6**). From this interface, the user can input the different parameters necessary to run OPTricluster**.** These input parameters are**:** the minimum number of genes in a cluster, the minimum number of samples in a cluster, and the ranking threshold.

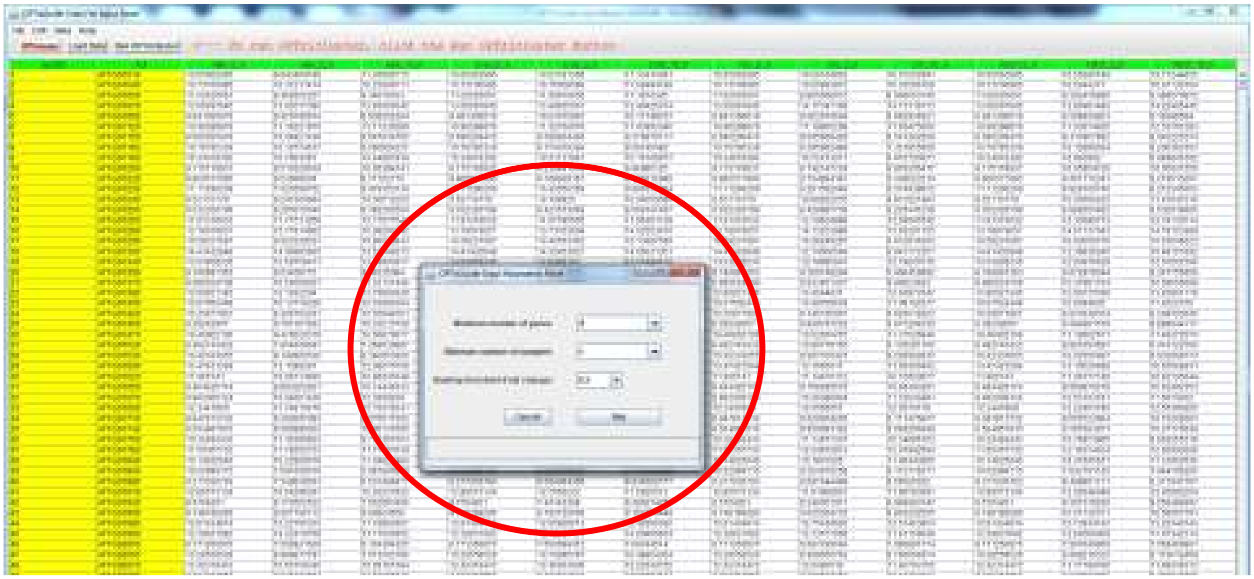

Figure 6: OPTricluster input parameters interface. It is used by the user to input the parameters necessary for running OPTricluster.

Once these input parameters are selected and validated, a new data table appears (**Figure 7**) in the *working space* of OPTricluster. In this new data table, new columns are added to the old ones, where each newly added column correspond to the ranking of the expression level of the genes across experimental time points in each sample.

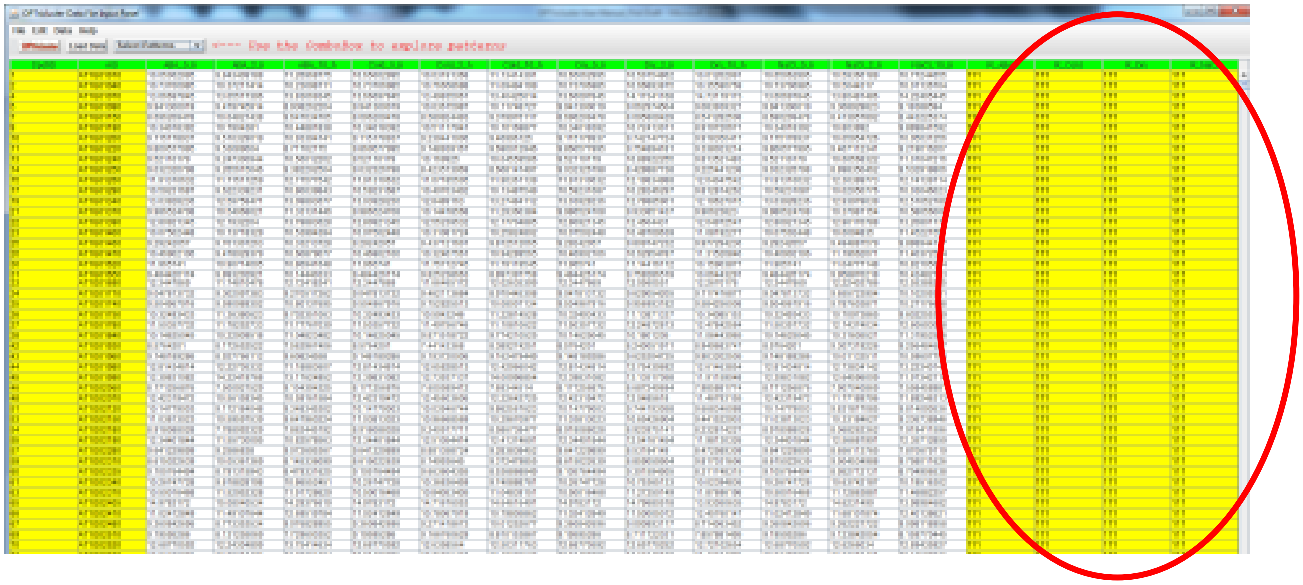

Figure 7: Example of the OPTricluster interface once input parameters are selected and validated. New columns are added. Each newly added column corresponds to the ranking of the expression level of the genes across experimental time points in each sample.

IV.3. Exploring OPTricluster patterns

Using the drop down menu (**Select Patterns**) from the *tool bar* (**Figure 8**), the user selects one of the following three types of patterns to explore: conserved, divergent, and constant.

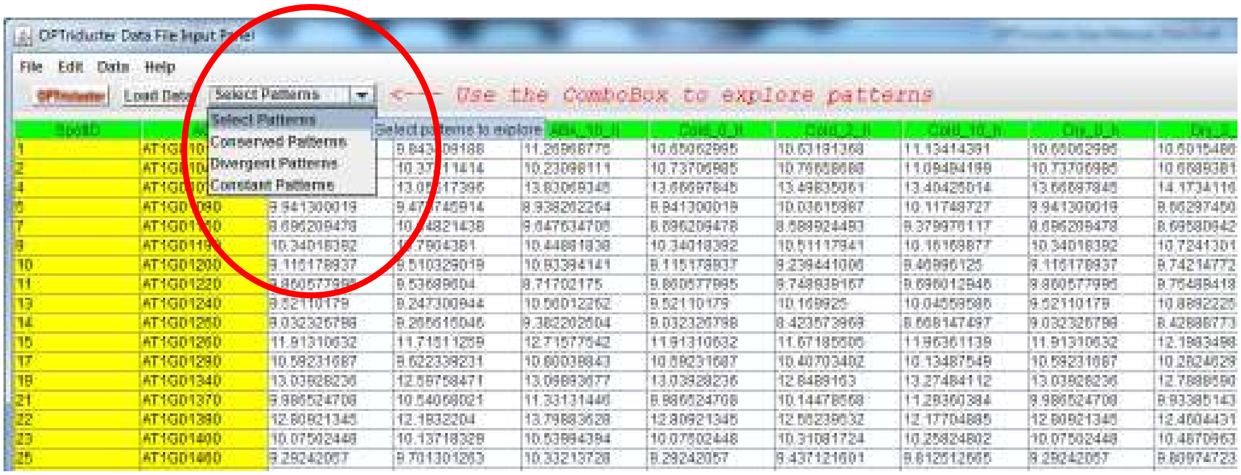

Figure 8: Example of the OPTricluster interface showing the **Select Patterns** drop down menu for OPTricluster patterns exploration.

IV.3.1 *Conserved patterns*

Conserved patterns correspond to group of genes having same behaviour across experimental time points in subsets of samples.

If **Conserved Patterns** are selected, then the *working space* of OPTricluster interface becomes **Figure 9**. The data table on the left corresponds to the input gene expression data with their ranking profile. The new table on the right corresponds to the conserved patterns. We will call this new table **Sample Table**. The fist column of the **Sample Table** corresponds to the subset of samples, the second column their description, the third the number of genes that are conserved in the corresponding subset of samples, the fourth column their percentage, and the fifth column are check boxes that can be selected and use to perform some other analysis on the selected conserved patterns.

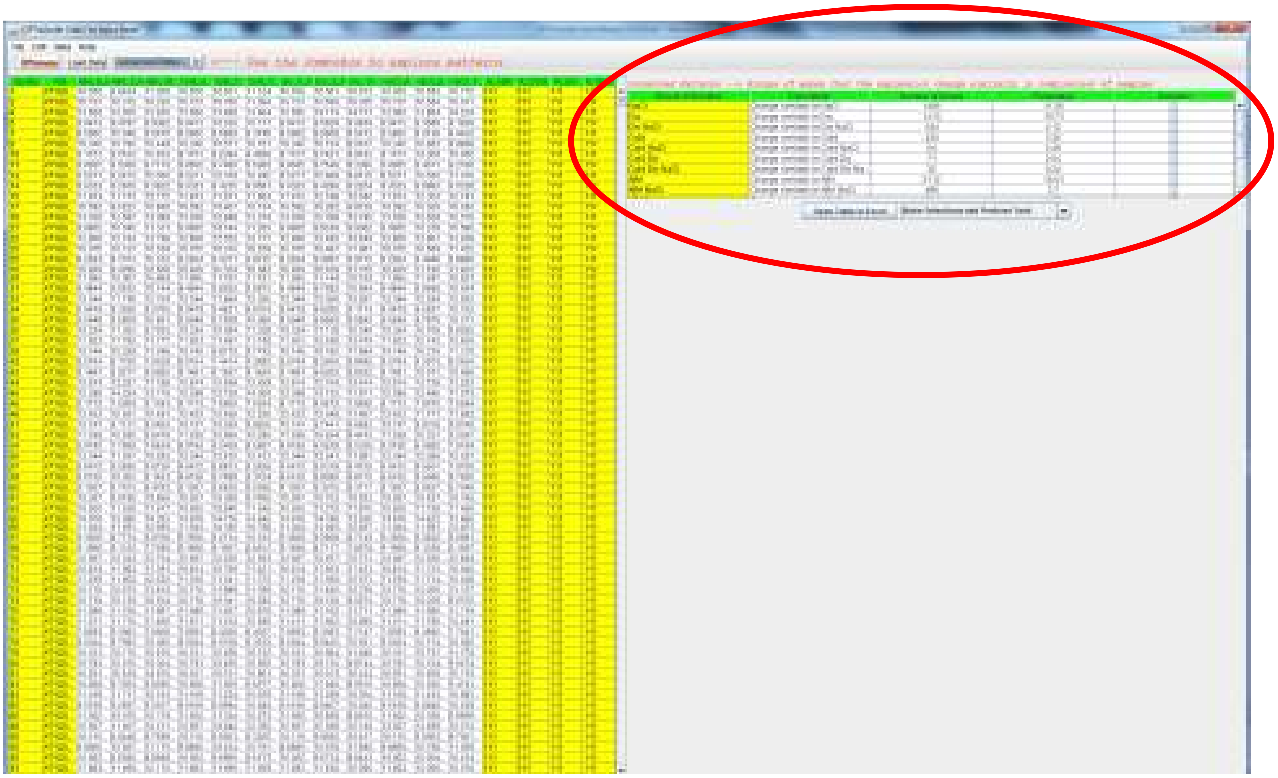

Figure 9: Example of the OPTricluster interface when a type of patterns (conserved patterns) to be explored is selected, showing the **Sample Table**.

Each cell of the column of the **Sample Table** that corresponds to the subset of samples is clickable. By double clicking (click twice) in one of these cells, a new data table appears below it (**Figure 10**). We call this new table **Ranking Table**. **Ranking Table** describes the set of ranking patterns, their percentage, and their statistical significance (p-values) computed using the methodology describes in [1].

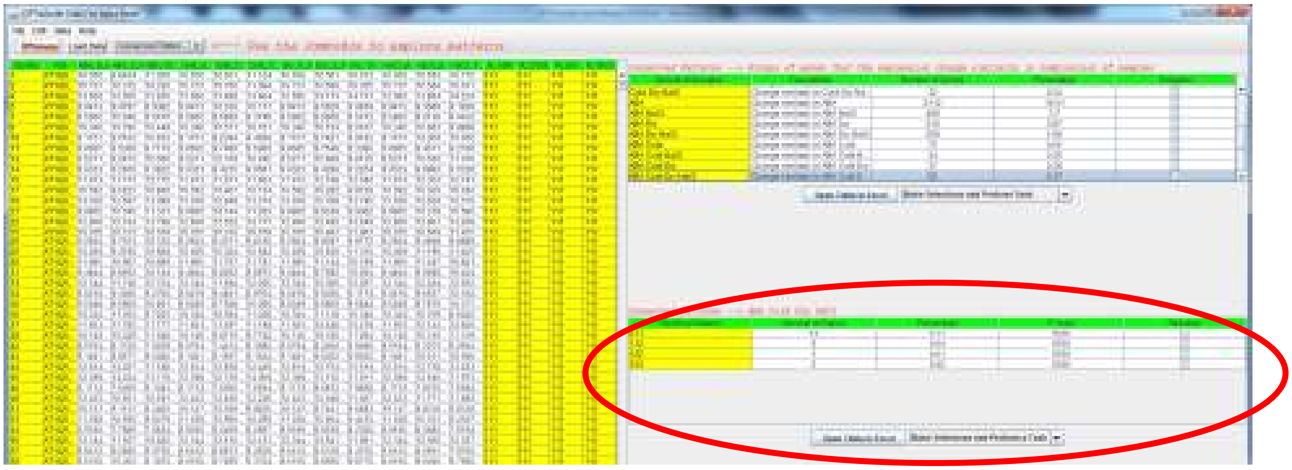

Figure 10: Example of the OPTricluster interface when a pattern to be explored is selected and a subset of sample selected (double clicking twice in a row of the **Sample Table**), showing the **Ranking Table**.

Furthermore, each cell of the first column of the **Ranking Table** is clickable. By double clicking (click twice) in one of these cells, a new table appears below it (**Figure 11**). This new data table is the **Cluster Table**. The **Cluster Table** describes the set of genes that belong to this group, their expression level, sample sets and time points.

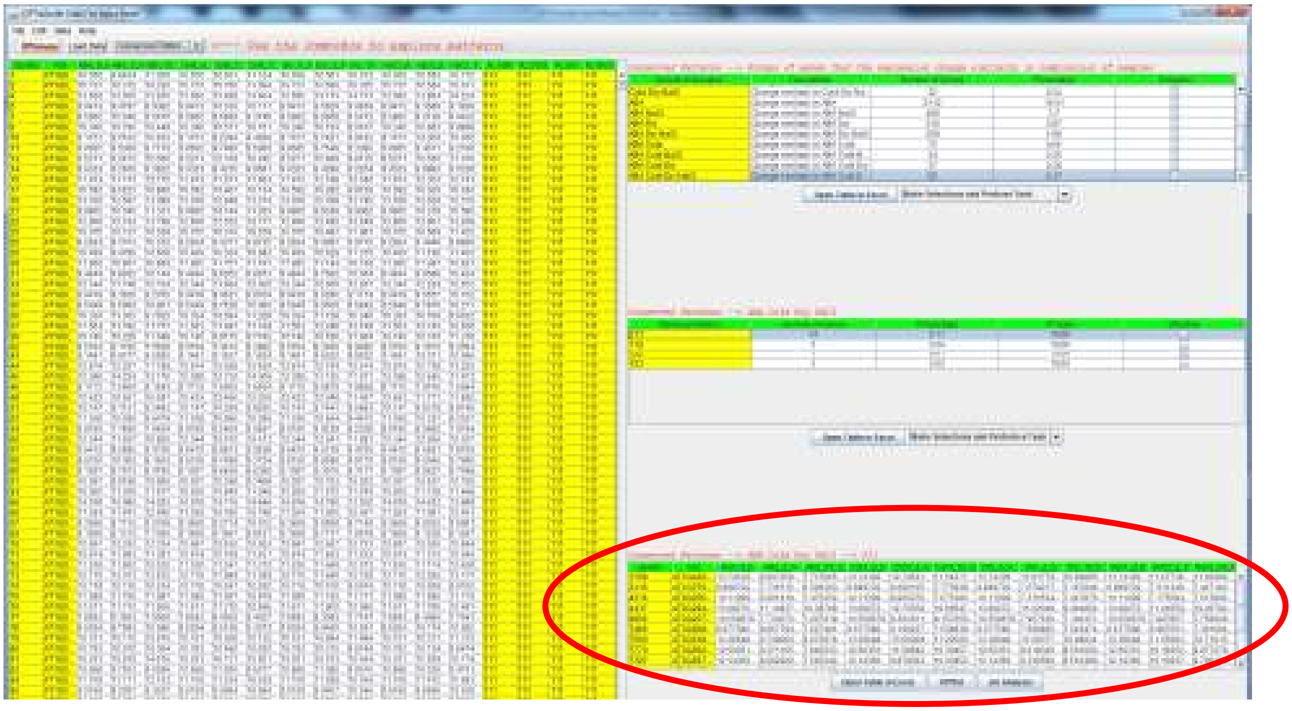

Figure 11: Example of the OPTricluster interface when a pattern to be explored is selected (Conserved Patterns Selected), a subset of sample selected (double clicking twice in a row of the **Sample Table**), and a ranking profile selected (double clicking twice in a row of the **Ranking Table**), showing the **Cluster Table**.

At each step along the way, via the "**Open Table in Excel**" button that appears under the **Sample Table** (**Figure 12**), **Ranking Table**, and the **Cluster Table**, the user can open the table in Excel and do more analysis in Excel using its rich capabilities.

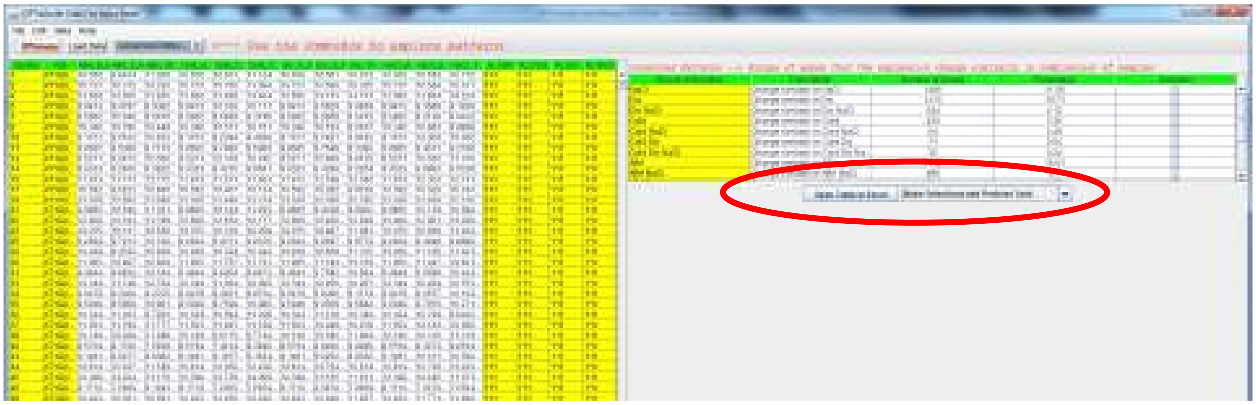

Figure 12: Additional OPTricluster commands that the user can exploit during the analysis to get more insights on the gene expression data.

The **Select Chart to Plot** drop down menu also allows the user to do more on the fly analyses of the data in the corresponding table (**Sample Table** and **Ranking Table**). These on the fly analyses are described in **Table 3**.

Table 3: **Select Chart to Plot** drop down menu description

**OPTricluster Explore Menu**	**Function**
Pie Chart	Plot the pie chart of the selected items
Pie Chart 3D	Plot the 3D pie chart of the selected items
Bar Chart	Plot the bar chart of the selected items
Bar Chart 3D	Plot the bar chart of the selected items
Difference	Take the difference of the selected items
GO Analysis	Gene Ontology analysis of the selected item
Open Selected in Excel	Open the expression level of the selected item in Excel
Merge (only in Ranking Table)	Merge the expression level of selected items

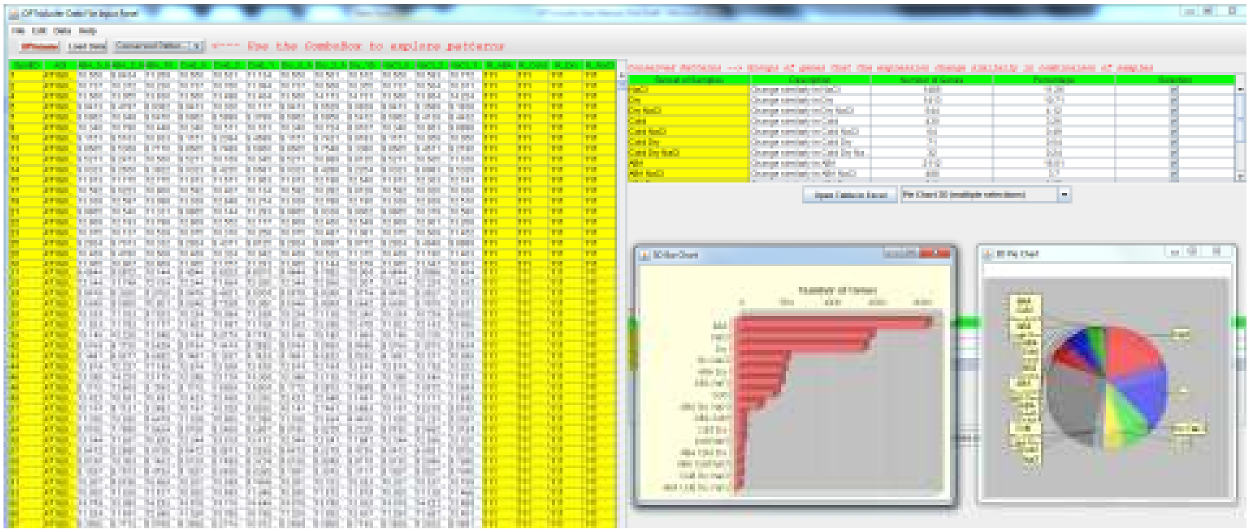

Figure 13: Example showing the plot of the Pie Chart and the Bar Chart representing the percentage of genes conserved in each selected subset of samples.

The **XYPlot** button located at the bottom of the **Cluster Table** allow the user to plot the expression level of genes in the 3D cluster selected, while the **GO Analysis** button allows the user to perform the gene ontology analysis of the selected cluster **Figure 14**.

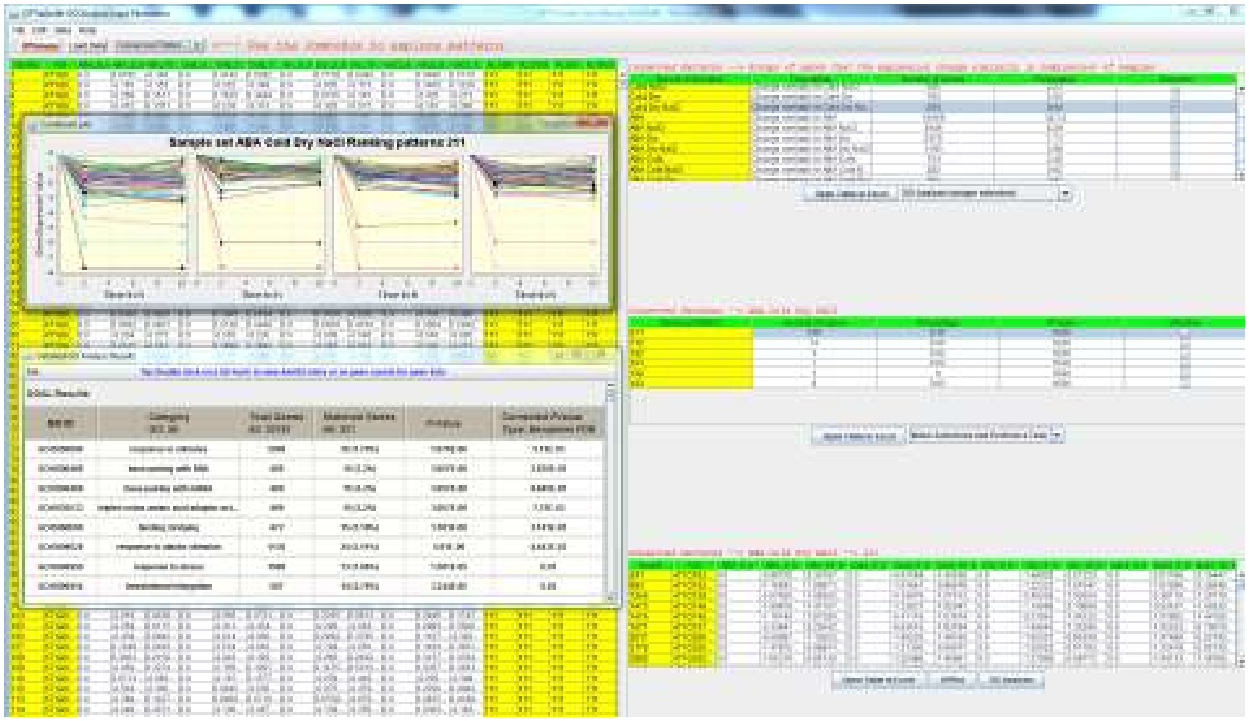

Figure 14: Plot of the expression profile (**XYPlot** button) of a cluster and its gene ontology analysis (**GO Analysis** button).

IV.3.2. *Divergent patterns*

Divergent patterns correspond to group of genes that behave differently in at least one sample along the time point experiments. Their exploration is similar to that of conserved patterns. This is done by selecting **Divergent Patterns** from the **Patterns Exploration** drop down menu. **Figure 15** shows and example of such patterns.

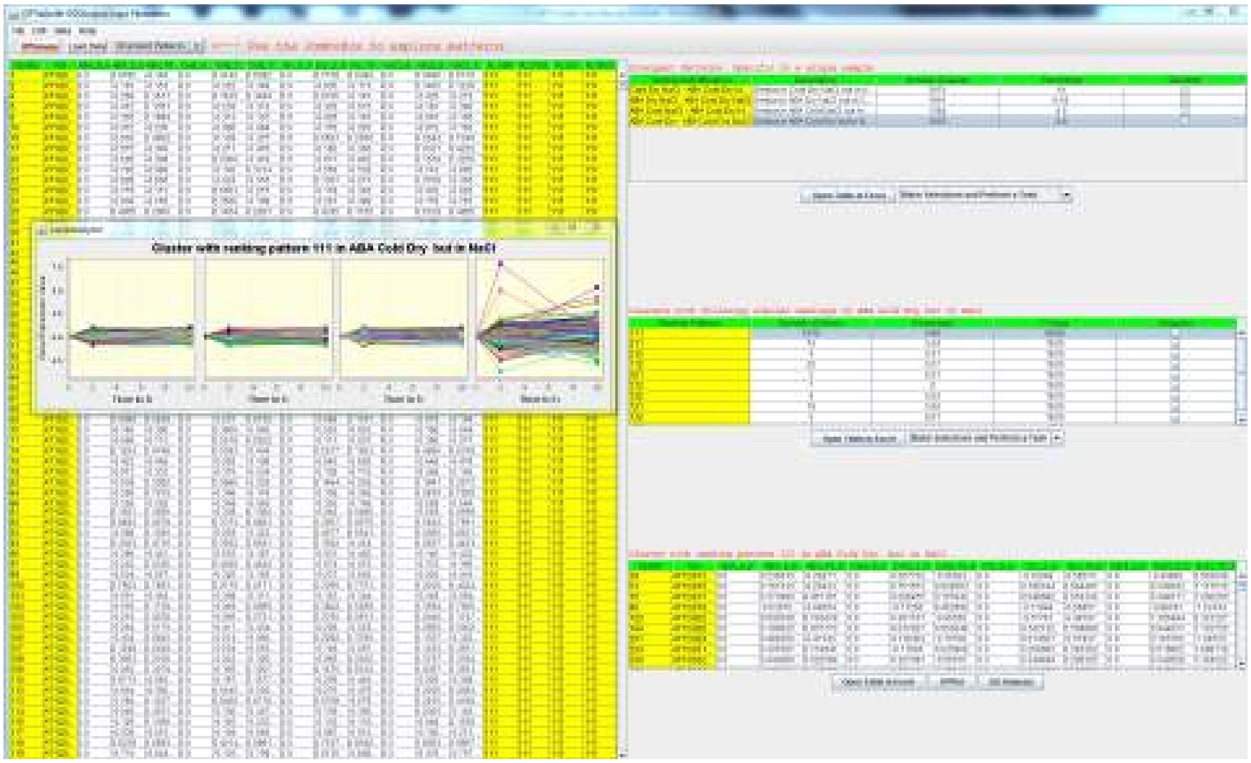

Figure 15: Example of divergent patterns exploration. The patterns are constant in the first three samples (first three chats), but different in the last one (last Chart).

IV.3.3. *Constant patterns*

Constant patterns are like conserved patterns, but unlike them, their expression level stay unchanged across experimental time points. Their exploration is carried out similarly to that of conserved patterns. This is done by selecting **Constant Patterns** from the **Patterns Exploration** drop down menu. **Figure 16** shows and example of such patterns.

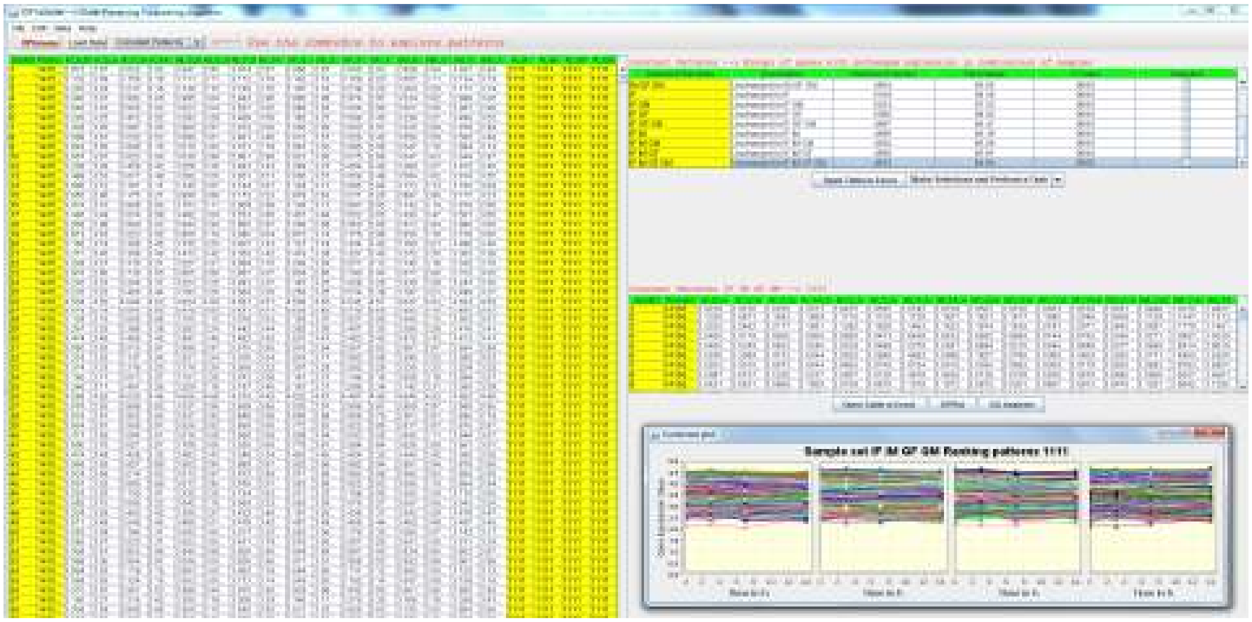

Figure 16: Example of constant patterns exploration. In this example, the patterns are unchanged in the four samples (four charts).

V. Integration to Gene Ontology (GO Analysis button)

In a post processing step, OPTricluster also makes use of external Gene Ontology files. OPTricluster can download the Gene Ontology and gene annotation files directly from the websites of the Gene Ontology [2]. This is done using the menu **Data → Update → Gene Ontology** for the ontology file, and **Data → Update → Species Annotation Files** for the species annotation files. This can also be done using the **Update Annotations** or the **Update Gene Ontology File** buttons located on the OPTricluster GO analysis input parameters interface (**Figure 17**).

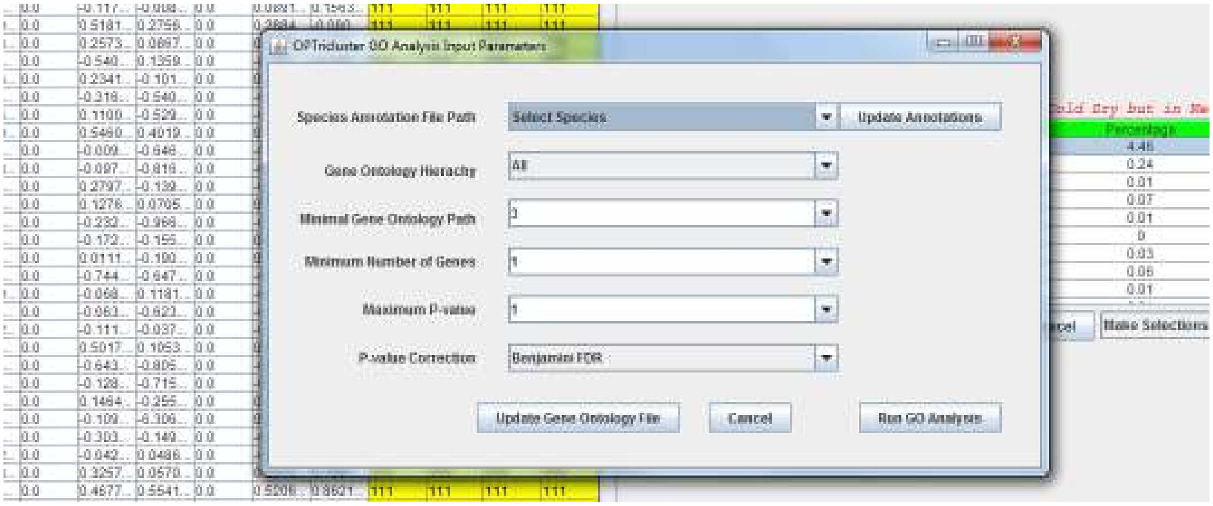

Figure 17: OPTricluster GO Analysis input parameters interface.

The **GO Analysis** button that appears at each step of the analysis allows the user to perform the gene ontology analysis of the current results. In fact the GO analysis plug-in of the Gene Ontology Analysis (GOAL) [3] package that we recently developed is integrated into OPTricluster for biological evaluation of the clusters. Thus the user can make use of the rich functionalities already integrated to the GOAL package to manipulate the GO results table **Figure 18**.

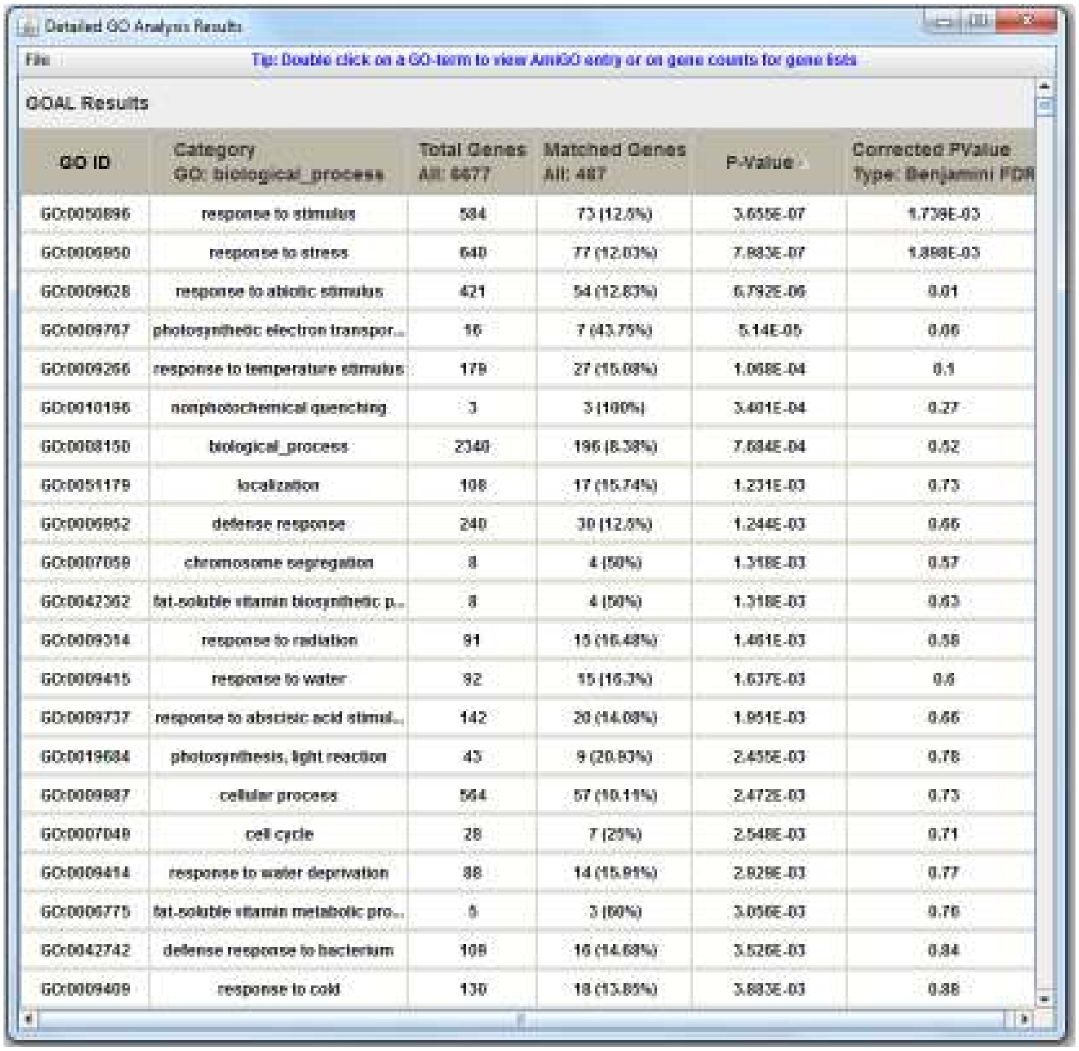

GO ID	Category GO: biological_process	Total Genes All: 6677	Matched Genes All: 487	P-Value	Corrected PValue Type: Benjamini FDR
GO:0050896	response to stimulus	584	73 (12.5%)	3.655E-07	1.739E-03
GO:0006950	response to stress	640	77 (12.03%)	7.983E-07	1.898E-03
GO:0009628	response to abiotic stimulus	421	54 (12.83%)	6.792E-06	0.01
GO:0009767	photosynthetic electron transpor...	16	7 (43.75%)	5.14E-05	0.06
GO:0009266	response to temperature stimulus	179	27 (15.08%)	1.068E-04	0.1
GO:0010196	nonphotochemical quenching	3	3 (100%)	3.401E-04	0.27
GO:0008150	biological_process	2340	196 (8.38%)	7.684E-04	0.52
GO:0051179	localization	108	17 (15.74%)	1.231E-03	0.73
GO:0006952	defense response	240	30 (12.5%)	1.244E-03	0.66
GO:0007059	chromosome segregation	8	4 (50%)	1.318E-03	0.57
GO:0042362	fat-soluble vitamin biosynthetic p...	8	4 (50%)	1.318E-03	0.63
GO:0009314	response to radiation	91	15 (16.48%)	1.461E-03	0.58
GO:0009415	response to water	92	15 (16.3%)	1.637E-03	0.6
GO:0009737	response to abscisic acid stimul...	142	20 (14.08%)	1.951E-03	0.66
GO:0019684	photosynthesis, light reaction	43	9 (20.93%)	2.455E-03	0.78
GO:0009987	cellular process	564	57 (10.11%)	2.472E-03	0.73
GO:0007049	cell cycle	28	7 (25%)	2.548E-03	0.71
GO:0009414	response to water deprivation	88	14 (15.91%)	2.929E-03	0.77
GO:0006775	fat-soluble vitamin metabolic pro...	5	3 (60%)	3.056E-03	0.76
GO:0042742	defense response to bacterium	109	16 (14.68%)	3.526E-03	0.84
GO:0009409	response to cold	130	18 (13.85%)	3.883E-03	0.88

Figure 18: Gene Ontology analysis results table. The user can exploit the functionalities already integrated to the GOAL software to manipulate the table. This could be through the file menu, or by double clicking in a cell GO term for example to see its description, or on gene count cell for the gene lists associated to the GO term.

VI. Integration to the JFreeChart Library

Portions of the interface of OPTricluster are implemented using the JFreeChart [4] library. This library is mostly used for graphing (Pie Chart, Bar Chart, XYPlot, etc...). The user can use all the

rich functionalities provided in JFreeChart to manipulate the charts. This is done by right clicking on the chart and exploring the chart using the dropped down menu **Figure 19**.

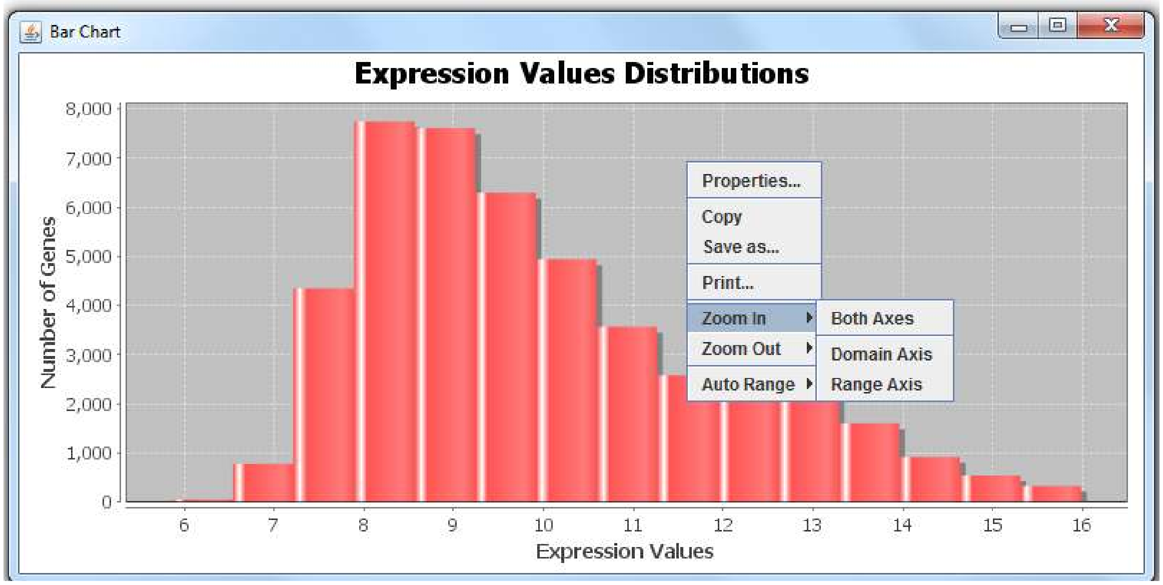

Figure 19: Manipulation of the JFreeChart charts by right clicking on the plot and exploiting the dropped down menu to manipulate the chart. This includes: changing the properties of the chart, copying, saving, printing, and zooming.

VII. References

1. Tchagang A.B, Phan S, Famili F, Shearer H, Fobert P, Huang Y, Zou J, Huang D, Cutler A, Liu Z, and Pan Y. Mining biological information from 3D short time-series gene expression data: the OPTricluster algorithm. *BMC Bioinformatics, u*nder review.
2. Gene Ontology [http://www.geneontology.org/]
3. Tchagang AB, Gawronski A, Bérubé H, Phan S, Famili F, Pan Y: GOAL: A Software Tool for Assessing Biological Significance of Genes group. *BMC Bioinformatics* 2010, 11:229.
4. JFreeChart [http://www.jfree.org/jfreechart/].
