# Supplementary figures and images for: Mining biological information from 3D short time-series gene expression data: the OPTricluster algorithm
Source: BMC Bioinformatics. 2012 Apr 4;13:54. doi: 10.1186/1471-2105-13-54 (PMC3376030; doi:10.1186/1471-2105-13-54)

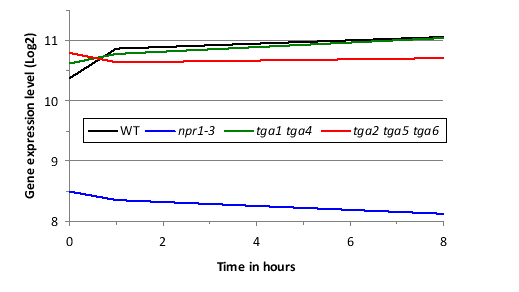

Supplement: Additional file 3 — Expression profile of NPR1 in different samples The axis corresponds to the time point experiments, the y-axis the expression level in Log2. Each curve corresponds to a sample. [file 1471-2105-13-54-S3.TIFF]

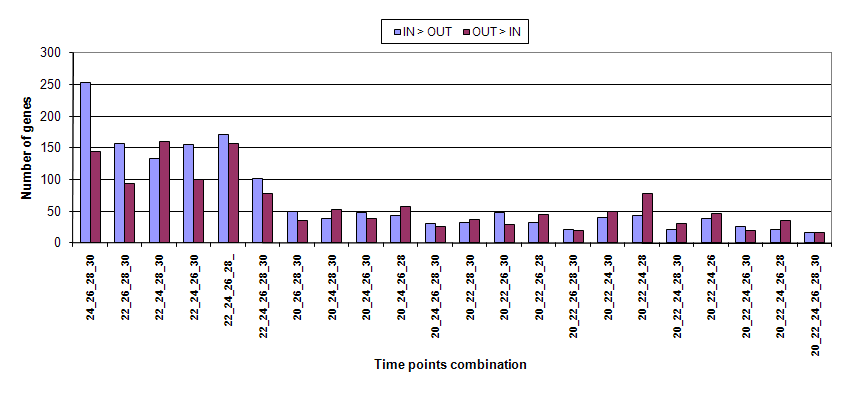

Supplement: Additional file 4 — Statistic of differences between inner and outer cotyledons The x-axis corresponds to the combination of time points, the y-axis the number of genes. [file 1471-2105-13-54-S4.TIFF]
