# Supplementary material for: Mining biological information from 3D short time-series gene expression data: the OPTricluster algorithm
Source: BMC Bioinformatics. 2012 Apr 4;13:54. doi: 10.1186/1471-2105-13-54 (PMC3376030; doi:10.1186/1471-2105-13-54)
Supplement: Additional file 5 — Gene Ontology analysis of whole seed Brassica napus clusters GO analysis of the 11 clusters in whole seed development Brassica napus. [file 1471-2105-13-54-S5.PPT]

## Slide 1
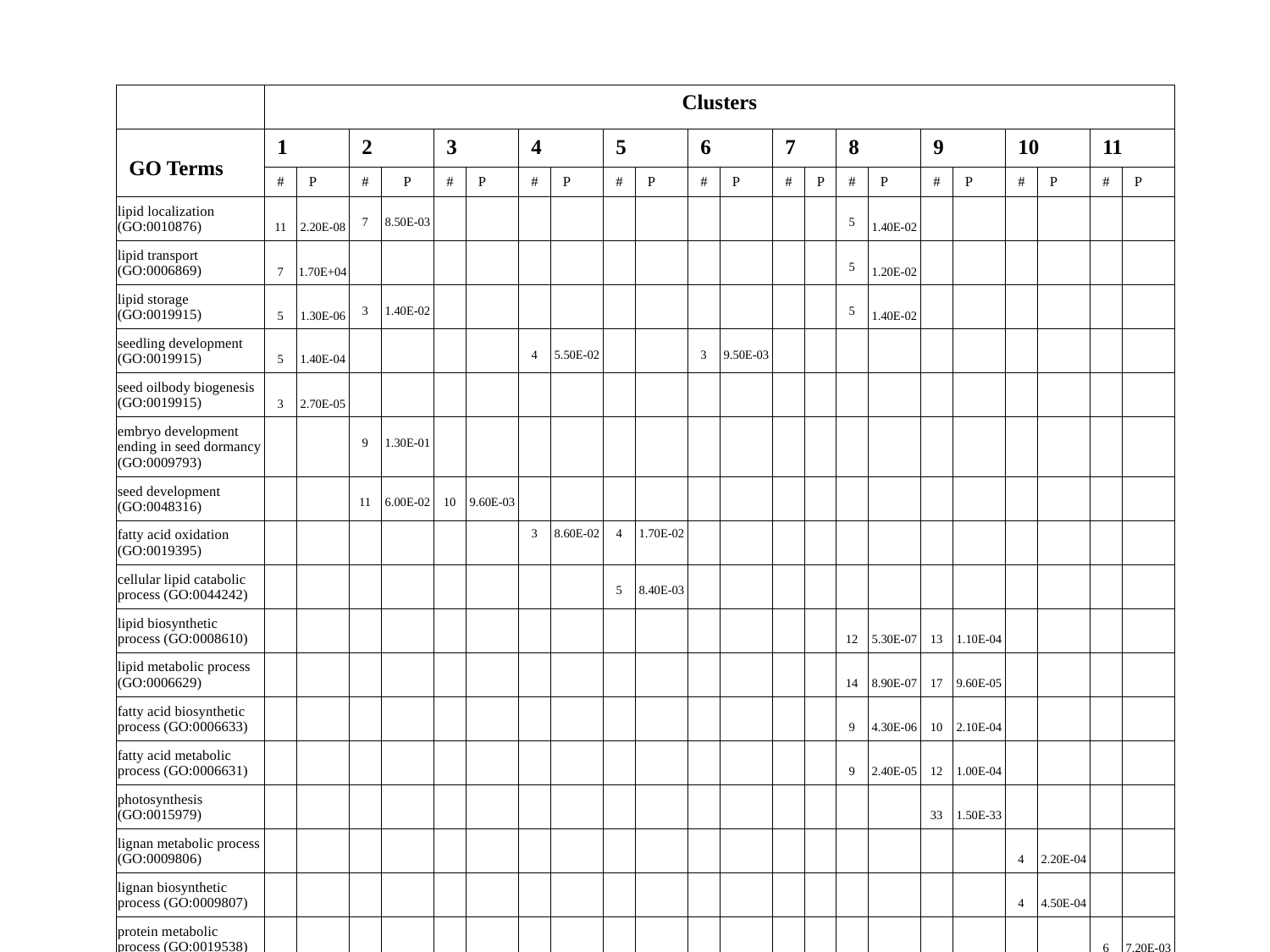

| | Clusters | | | | | | | | | | | | | | | | | | | | | |
| --- | --- | --- | --- | --- | --- | --- | --- | --- | --- | --- | --- | --- | --- | --- | --- | --- | --- | --- | --- | --- | --- | --- |
| GO Terms | 1 | | 2 | | 3 | | 4 | | 5 | | 6 | | 7 | | 8 | | 9 | | 10 | | 11 | |
| | # | P | # | P | # | P | # | P | # | P | # | P | # | P | # | P | # | P | # | P | # | P |
| lipid localization (GO:0010876) | 11 | 2.20E-08 | 7 | 8.50E-03 | | | | | | | | | | | 5 | 1.40E-02 | | | | | | |
| lipid transport (GO:0006869) | 7 | 1.70E+04 | | | | | | | | | | | | | 5 | 1.20E-02 | | | | | | |
| lipid storage (GO:0019915) | 5 | 1.30E-06 | 3 | 1.40E-02 | | | | | | | | | | | 5 | 1.40E-02 | | | | | | |
| seedling development (GO:0019915) | 5 | 1.40E-04 | | | | | 4 | 5.50E-02 | | | 3 | 9.50E-03 | | | | | | | | | | |
| seed oilbody biogenesis (GO:0019915) | 3 | 2.70E-05 | | | | | | | | | | | | | | | | | | | | |
| embryo development ending in seed dormancy (GO:0009793) | | | 9 | 1.30E-01 | | | | | | | | | | | | | | | | | | |
| seed development (GO:0048316) | | | 11 | 6.00E-02 | 10 | 9.60E-03 | | | | | | | | | | | | | | | | |
| fatty acid oxidation (GO:0019395) | | | | | | | 3 | 8.60E-02 | 4 | 1.70E-02 | | | | | | | | | | | | |
| cellular lipid catabolic process (GO:0044242) | | | | | | | | | 5 | 8.40E-03 | | | | | | | | | | | | |
| lipid biosynthetic process (GO:0008610) | | | | | | | | | | | | | | | 12 | 5.30E-07 | 13 | 1.10E-04 | | | | |
| lipid metabolic process (GO:0006629) | | | | | | | | | | | | | | | 14 | 8.90E-07 | 17 | 9.60E-05 | | | | |
| fatty acid biosynthetic process (GO:0006633) | | | | | | | | | | | | | | | 9 | 4.30E-06 | 10 | 2.10E-04 | | | | |
| fatty acid metabolic process (GO:0006631) | | | | | | | | | | | | | | | 9 | 2.40E-05 | 12 | 1.00E-04 | | | | |
| photosynthesis (GO:0015979) | | | | | | | | | | | | | | | | | 33 | 1.50E-33 | | | | |
| lignan metabolic process (GO:0009806) | | | | | | | | | | | | | | | | | | | 4 | 2.20E-04 | | |
| lignan biosynthetic process (GO:0009807) | | | | | | | | | | | | | | | | | | | 4 | 4.50E-04 | | |
| protein metabolic process (GO:0019538) | | | | | | | | | | | | | | | | | | | | | 6 | 7.20E-03 |
